# HvPap-1 C1A Protease Participates Differentially in the Barley Response to a Pathogen and an Herbivore

**DOI:** 10.3389/fpls.2017.01585

**Published:** 2017-09-12

**Authors:** Mercedes Diaz-Mendoza, Blanca Velasco-Arroyo, M. Estrella Santamaria, Isabel Diaz, Manuel Martinez

**Affiliations:** ^1^Centro de Biotecnologia y Genomica de Plantas, Universidad Politecnica de Madrid – Instituto Nacional de Investigacion y Tecnologia Agraria y Alimentaria Madrid, Spain; ^2^Departamento de Biotecnologia-Biologia Vegetal, Escuela Tecnica Superior de Ingenieria Agronomica, Alimentaria y de Biosistemas, Universidad Politecnica de Madrid Madrid, Spain

**Keywords:** plant–pathogen interaction, plant–herbivore interaction, *Hordeum vulgare*, *Tetranychus urticae*, *Magnaporthe oryzae*, cysteine protease

## Abstract

Co-evolutionary processes in plant–pathogen/herbivore systems indicate that protease inhibitors have a particular value in biotic interactions. However, little is known about the defensive role of their targets, the plant proteases. C1A cysteine proteases are the most abundant enzymes responsible for the proteolytic activity during different processes like germination, development and senescence in plants. To identify and characterize C1A cysteine proteases of barley with a potential role in defense, mRNA and protein expression patterns were analyzed in response to biotics stresses. A barley cysteine protease, *HvPap-1*, previously related to abiotic stresses and grain germination, was particularly induced by flagellin or chitosan elicitation, and biotic stresses such as the phytopathogenic fungus *Magnaporthe oryzae* or the phytophagous mite *Tetranychus urticae*. To elucidate the *in vivo* participation of this enzyme in defense, transformed barley plants overexpressing or silencing *HvPap-1* encoding gene were subjected to *M. oryzae* infection or *T. urticae* infestation. Whereas overexpressing plants were less susceptible to the fungus than silencing plants, the opposite behavior occurred to the mite. This unexpected result highlights the complexity of the regulatory events leading to the response to a particular biotic stress.

## Introduction

Plant proteases are key enzymes involved in protein degradation mechanisms associated to numerous physiological processes (van der Hoorn, 2008). Among the 17 families of cysteine proteases (CysProt) currently identified in plants (MEROPS database, Rawlings et al., 2016), members of the C1A family of papain-like proteases are the most abundant. These proteases are classified as cathepsin L-, B-, H- and F-like according to their gene structures and phylogenetic relationships (Martinez and Diaz, 2008; Richau et al., 2012). Physiologically, C1A proteases have widely been implicated in endogenous processes, such as senescence, abscission, programmed cell death, fruit ripening and mobilization of proteins accumulated in seeds and tubers (Grudkowska and Zagdanska, 2004; Martinez et al., 2012). Besides, the expression of CysProt genes is enhanced under various abiotic stresses, which trigger reorganization of metabolism, remodeling of cell protein components, degradation of damaged or unnecessary proteins and nutrient remobilization along leaf senescence events (Diaz-Mendoza et al., 2014, 2016; Velasco-Arroyo et al., 2016). Likewise, C1A CysProt play crucial roles in the response of the plant to different biotic stresses related to pathogen perception, disease resistance signaling, and defense against insects (van der Hoorn and Jones, 2004; Shindo and van der Hoorn, 2008; Misas-Villamil et al., 2016).

There are some examples of plant CysProt induced by fungal, bacterial and viral infection that have been directly associated to plant resistance. Cathepsin B genes from *Nicotiana* and *Arabidopsis* are involved in pathogen responses and are required for full basal resistance against different bacteria (Gilroy et al., 2007; McLellan et al., 2009). Similarly, silencing of the CysProt C14 of tomato and potato led to an increased susceptibility to *Phytophthora infestans* (Kaschani et al., 2010; Bozkurt et al., 2011); the lack of the ortholog protein in *Arabidopsis*, RD21, also provoked the same effect toward to the fungus *Botrytis cinerea* whereas the growth of *Sclerotina sclerotiorum* was compromised in plants lacking RD21 (Shindo et al., 2012; Lampl et al., 2013). Besides, overexpression of *AcCP2* gene, a C1A CysProt from pineapple fruit, improves the resistance to the fungal pathogen *B. cinerea* in *Arabidopsis* (Wang et al., 2014), and silencing of the *Arabidopsis* KDEL CysProt, AtCEP1, leads to a higher susceptibility to the fungus *Erysiphe cruciferarum* (Höwing et al., 2014). Finally, tomato yellow leaf curl geminivirus inhibits the host CYP1 protein, a CysProt involved in plant defense against diverse pathogens (Bar-Ziv et al., 2012, 2015). On the other hand, it was documented that biotic stresses mediated by pathogens induce senescence symptoms such as proteolysis and nutrient mobilization (Pogány et al., 2015). Some senescence associated genes induced during plant aging were also observed during pathogen infections (Pontier et al., 1999). Some evidence supports the connection of these two processes. For example, down-regulation of *OsSAG12-1* resulted in earlier senescence and enhanced cell death in transgenic rice plants infected by *Xanthomonas oryzae* (Singh et al., 2013).

The role of some CysProt in plant defense against herbivores has also been documented (Shindo and van der Hoorn, 2008). CysProt like papain from pineapple showed toxicity to lepidopteran larvae, which had a reduced weight when fed with leaves containing papain (Konno et al., 2004). A 33 kDa CysProt from maize, Mir1 (Maize inbred resistance 1), accumulates in response to caterpillars. Larvae fed with transgenic maize callus overexpressing the *Mir1* gene were significantly smaller than those fed with callus from control plants (Pechan et al., 2000, 2002). There are also some examples of proteases involved in leaf senescence linked to herbivore defense. The senescence associated gene *SAG12* was induced in *Arabidopsis* plants by infestation of *Bemisia tabaci* (Kempema et al., 2015). According to their different life styles, plant pathogens can be divided into biotrophs, which prefer living cells for nutritional purposes, and necrotrophs, which select dead cells (Glazebrook, 2005). Hemibiotrophic fungi, such as *M. oryzae*, combine both lifestyles (Fernandez and Wilson, 2012). *M. oryzae* is a fungal pathogen of rice but it is also able to infect other cereals, including barley (Hyon et al., 2012). Thus, *M. oryzae* and barley can be used as a model system for the analysis of interactions between fungal pathogens and small grain cereals at the molecular level (Tanaka et al., 2010; Ulferts et al., 2015).

Herbivores injure plant tissues with their different feeding methods. This damage is perceived by the plant, which starts mobilizing defense signaling pathways (Santamaria et al., 2013; Acevedo et al., 2015). Numerous herbivore elicitors and effectors have been identified, but the characterization of plant receptors that perceive this herbivore signaling is still limited (Acevedo et al., 2015). The two-spotted spider mite *T. urticae* is one of the most polyphagous arthropods and it represents an important pest in crop plants, including barley (Migeon and Dorkeld, 2006–2017). *T. urticae* feeding mode consists of stylet penetrating parenchyma cells and sucking their contents (Bensoussan et al., 2016), a mechanism that has evinced the activation of defense pathways in several plant species (Kant et al., 2004; Zhurov et al., 2014; Martel et al., 2015; Diaz-Riquelme et al., 2016). The genome of *T. urticae* has been sequenced and a broad range of tools and protocols have been developed to study its interaction with the plant (Grbić et al., 2011; Santamaria et al., 2012; Cazaux et al., 2014). Therefore, the two-spotted spider mite is suitable to be used as a model for plant-herbivore interaction studies.

The barley C1A CysProt family members were previously identified and their transcriptional response against abiotic stresses characterized (Diaz-Mendoza et al., 2014; Velasco-Arroyo et al., 2016). In this work we have used the pathogen *M. oryzae* and the herbivore *T. urticae* to analyze the role of C1A CysProt in the barley response to different biotic stresses. Besides, we have determined the specific role in the defense mechanisms against pathogens or herbivores of barley HvPap-1, a C1A CysProt previously associated to endogenous processes and the response to abiotic stresses.

## Materials and Methods

### Plant Material and Growth Conditions

Barley plants of *Hordeum vulgare* cv. Golden Promise were used. Grains were germinated in soil and grown at 22°C under a 16 h light/8 h darkness photoperiod for 7 days in Sanyo MLR-350-H chambers. Barley transgenic lines overexpressing or silencing the *HvPap-1* gene (OE Pap1 and KD Pap1, respectively) were generated using *Agrobacterium*-mediated gene transfer and haploid technology in collaboration with Dr. Jochen Kumlehn’s group (IPK-Gatersleben, Germany) as described in Diaz-Mendoza et al. (2016). The homozygous transgenic barley lines for the inserted constructions were validated by molecular and biochemical characterization of HvPap-1, including copy number, and mRNA and protein expression patterns as described in Diaz-Mendoza et al. (2016). Transgenic barley lines were grown at the same conditions described above.

### Elicitor Treatments

The flagellin (flg22) peptide (amino acid sequence QRLSTGSRINSAKDDAAGLQIA) was synthesized by AnaSpec laboratories at a purity level of ≥95% (v/v). Stocks were prepared by dissolving the peptide in H_2_O at a concentration of 10 mM, stored at -20°C, and subsequently diluted in H_2_O to a 5 μM concentration for experiments. Chitosan solutions were prepared by dissolving hydrolyzed chitosan purified from crab shells (Sigma–Aldrich) in 0.02% (w/v) acetic acid at a concentration of 100 μg/mL. All elicitor solutions were applied as a foliar spray over wild-type 7-day-old barley leaves. Three pots of two plants each were used per treatment and three independent experiments were performed. The solvent used to solubilize elicitors (H_2_O or 0.02% (v/v) acetic acid, for fgl22 or chitosan, respectively) was used for mock treatments. Plants were further incubated at the same growth conditions described earlier. Barley leaves were monitored at different time points. Finally, leaves were harvested after 24 h of elicitor treatments. These samples were frozen into liquid nitrogen and stored at -80°C for further analysis.

### *Magnaporthe oryzae* Infections

As example of biotic stress mediated by pathogens, 7-day-old barley plants of wild-type and transgenic lines overexpressing or silencing the *HvPap-1* gene were infected with the fungus *M. oryzae*. For infection assays, the fungal isolate used in this study was the *M. oryzae* wild-type strain Guy11 (Leung et al., 1988), kindly provided by Dr. Sesma, CBGP-UPM-INIA, Madrid. The growth, maintenance and media composition of *M. oryzae* were as previously described in Tucker et al. (2010). Infection assays were performed in whole plant leaves by spray inoculations using an airbrush nebulizer compressor as described in Sesma and Osbourn (2004). Seven plants were used per treatment and three independent experiments were performed. Each pot was sprayed with 1 mL suspension of 10^5^ conidia/mL counted in a Neubauer counting chamber, in 0.25% (v/v) gelatin, or just gelatin in the case of control treatments. Plants were covered by a plastic bag to avoid conidia dispersion and the same confinement system was applied to the controls. The plants were further incubated at 25°C, 65% RH, under a 16 h light/8 h darkness photoperiod. Barley leaves were monitored at different time points to score disease symptoms. Leaves were harvested after 3 and 7 days post inoculation (dpi). This material was imaged and scanned, or frozen in liquid nitrogen and stored at -80°C for further analysis.

### *Tetranychus urticae* Infestations

A colony of the two-spotted spider mite *T. urticae* London strain (Acari: Tetranychidae), provided by Dr. Miodrag Grbic (UWO, Canada), was maintained on beans in a Sanyo MLR-350-H growth chamber at 25°C under a 16 h light/8 h darkness photoperiod. This colony was transferred to barley where it was maintained under the same conditions for more than 30 generations to ensure host adaptation To induce biotic stresses mediated by pest attack, 7-day-old barley plants, wild-type and transgenic lines overexpressing or silencing the *HvPap-1* gene, were infested with 20 barley-adapted adults of *T. urticae* per plant. Seven plants were used per treatment and three independent experiments were performed. Barley plants were confined in independent pots with a plastic cylinder covered on top by nylon nets to avoid dispersion of mites and the same isolation system was applied to control plants. Plants were further incubated at 25°C under a 16 h light/8 h darkness photoperiod. Barley leaf damage was monitored at different time points after spider mite feeding. Leaves were harvested after 7 and 14 days of mite treatment. Samples were imaged and scanned, or frozen into liquid nitrogen and stored at -80°C for further analysis.

### Damage Quantification Assays

*Magnaporthe oryzae* and *Tetranychus urticae* lesions observed on barley leaves were scanned using a scanner hp scanjet (HP Scanjet 5590 Digital Flatbed Scanner) and foliar damage on transformed and non-transformed lines after treatments was analyzed. Damaged leaf surface area was measured using the Fiji-ImageJ software (Schindelin et al., 2012) and was quantified as mm^2^ of injured area. Seven replicates per line were analyzed and three independent experiments were performed.

### Real-Time Reverse Transcription Quantitative PCR Analyses (RT-qPCR)

For real-time RT-qPCR studies, total RNA was extracted from frozen barley leaves by the phenol/chloroform method, followed by precipitation with 8 M LiCl (Oñate-Sánchez and Vicente-Carbajosa, 2008) and digested with DNase (Promega). cDNAs were synthesized from 2 μg of RNA using the RevertAid H Minus First Strand cDNA Synthesis Kit (Thermo Scientific) following the manufacturer’s instructions. RT-qPCR analyses were performed for triplicated samples by means of a CFX96 Real-time system (BioRad) using SYBR Green (Roche) as a detection system. Protease mRNA quantification was expressed as 2^∧-dCt^ (Livak and Schmittgen, 2001) and normalized to barley cyclophilin (*HvCycl* gene) mRNA levels (Hua et al., 2015). Expression levels of the *M. oryzae* small subunit of ribosomal RNA (Mo28S-rRNA) (Marcel et al., 2010) and *T. urticae* Ribosomal Protein 49 (TuRP49) (Morales et al., 2016) were quantified as 2^∧-dCt^ by subtracting the dC_t_ value of the barley cyclophilin from the dC_t_ value of the fungal or mite probes to normalize for the amount of barley tissue present in each sample. Fold change values were expressed using the ddC_t_ method (Livak and Schmittgen, 2001) relative to the normalized expression of the same genes in non-treated plants. The primers used are shown in Supplementary Table 1. Efficiency of the primers was determined based on the slope of a standard curve and was between 95 and 105% for all primer pairs tested.

### Western-Blot Analyses

Total protein was extracted from treated and control barley plants by grinding leaf tissue in liquid nitrogen before the addition of 500 μl of extraction buffer (150 mM NaCl, 50 mM sodium phosphate, pH 6 and 2 mM EDTA). After centrifugation at 15,600 × *g* for 10 min at 4°C, the supernatant was used for protein quantification according to the method of Bradford (1976), with the bovine serum albumin as a standard. After separation on SDS-polyacrylamide gels (12–15%, w/v) according to Laemmli (1970), proteins were electro-transferred onto nitrocellulose membrane (GE Healthcare) and blocked in PBS (137 mM NaCl, 27 mM KCl, 10 mM Na_2_HPO_4_, 2 mM KH_2_PO_4_ pH 7.4) antisera buffer containing 5% (w/v) powdered skim milk, for 1 h. Immunoblotting was performed as described in Diaz-Mendoza et al. (2016), with anti-peptide polyclonal antibodies specifically selected against each protease whose sequences are indicate in Supplementary Table 2. All protease antibodies were produced in rabbits by Pineda Antibody Services. Polyclonal antibody against the Large Subunit of Rubisco (anti-LSR) was supplied by Agrisera. Optimal dilutions of primary antibodies were used according to Diaz-Mendoza et al. (2016).

### RNA Isolation, cDNA Library Construction and Illumina Sequencing

Total RNA was isolated from barley leaves as described above. Using poly-T oligo-attached magnetic beads, mRNAs were purified from the total RNA. Then, the mRNAs were fragmented and cDNA was synthesized using random hexamer-primers, DNA polymerase I and RNase H. The double-stranded cDNAs were purified with magnetic beads and ligated to adaptors for Illumina sequencing. The quality and quantity of the library was verified using an Agilent 2100 Bioanalyzer and an ABI StepOnePlus Real-Time PCR system, respectively. The cDNA libraries were sequenced using the Illumina HiSeq2000 platform by the Beijing Genomics Institute (BGI). More than 10 M single-end reads were obtained for each sample (three biological replicates).

### Sequence Data Analysis and Annotation

Raw reads in fastq format were firstly filtered and reads with adaptor sequences and low quality reads were removed. The gene and genome sequences of *H. vulgare* retrieved from the PGSB/MIPS PlantsDB website^1^ (Nussbaumer et al., 2013) were used as the reference databases (International Barley Genome Sequencing Consortium, 2012). Two different approaches were performed: (i) all the clean reads were mapped to the reference genome using SOAPAligner/SOAP2 (Li et al., 2009). The transcripts abundance was normalized by the RPKM (reads per kilobase of exon per million reads) algorithm (Mortazavi et al., 2008). Differentially expressed genes (DEGs) between groups were obtained using the NOISeq method (Tarazona et al., 2011) with a log2Ratio (fold change) higher than 1 and a probability of differential expression higher than 0.8; and (ii) all the clean reads were pseudoaligned to the reference High Confidence genes using Kallisto (Bray et al., 2016). The transcript abundance was quantified as TPM (transcripts per million) and 100 bootstrap samples were performed. DEGs between groups were obtained using the Wald test of the Sleuth method (Pimentel et al., 2016) with a b ratio (bias) higher than 1 and a *q*-value (false positive probability) lower than 0.001. Venn diagrams were created by the Venny 2.1 utility^2^ (Oliveros, 2007–2015) and heatmaps were performed by the shinyHeatmaply application^3^. Gene enrichment analyses were performed with the Fischer’s exact test using topGO package in R^4^ and the GO file retrieved from the PGSB/MIPS PlantsDB website.

### Statistical Analysis

For statistical analysis, the expression of individual C1A CysProt genes in WT background was compared between control and elicitor/biotic stimuli samples. Foliar damage and individual C1A CysProt expression data were compared among WT and transformed lines in control conditions or after biotic treatment. All analyses were performed by one-way ANOVA, followed by t-student or Student Newman-Keuls (SNK) tests using the statistic software R Project (v.3.1.2) package. In figures where pairwise samples were compared, one or two asterisks indicated significant differences (*t*-test, *P* < 0.01 and *P* < 0.001, respectively). In figures where more than two samples were compared, different letters indicated significant differences (SNK test, *P* < 0.01).

## Results

### Expression of C1A Proteases Is Modified in Barley Leaves during the Response to Elicitors

To check the transcriptional responses of C1A CysProt against biotic stresses, three cathepsin L-like (*HvPap-4*, *-6* and *-16*), one cathepsin F-like (*HvPap-1*), one cathepsin H-like (*HvPap-12*) and one cathepsin B-like (*HvPap-19*) genes were selected, all of them previously studied under severe senescence processes induced by continuous darkness and nitrogen deprivation (Velasco-Arroyo et al., 2016).

**FIGURE 1:**
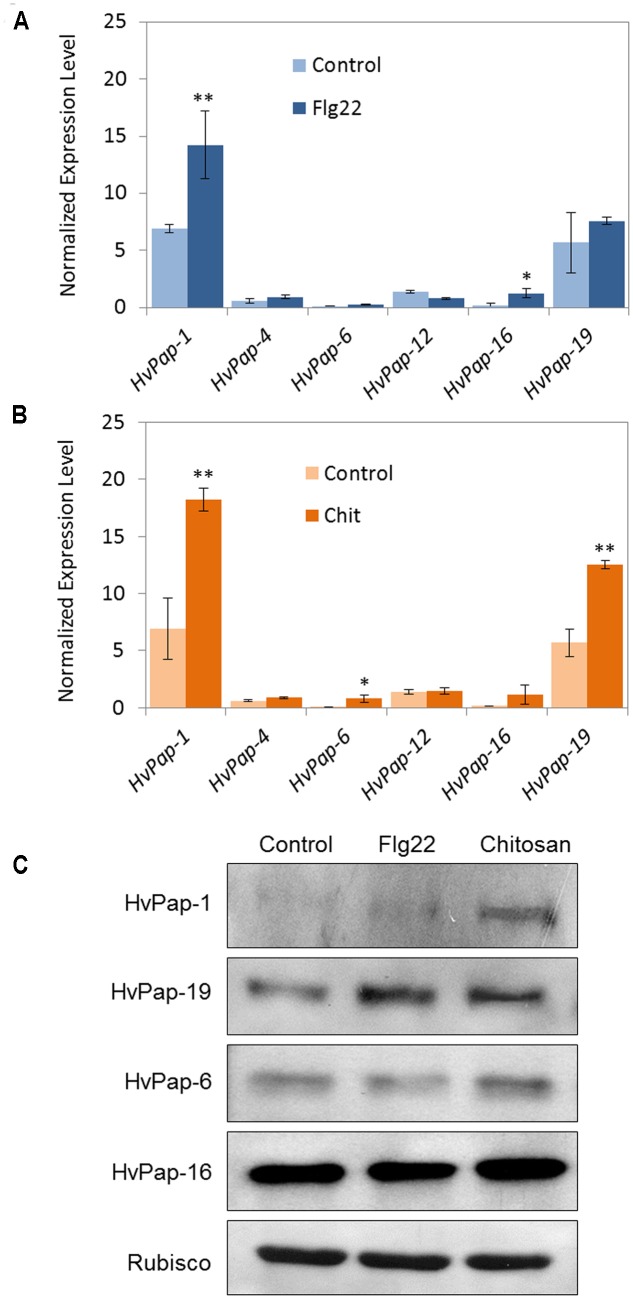
mRNA expression levels of barley C1A CysProt genes after 24 h of elicitor treatments. Expression of barley CysProt genes (*HvPap-1, -4, -6, -12, -16* and *-19*) in leaves treated with **(A)** flagellin (Flg22) and **(B)** chitosan (Chit), controls (light colors) and treated (dark colors). Data were determined by RT-qPCR and expressed as mRNA levels of C1A CysProt genes normalized to barley *cyclophilin* mRNA content. One (^∗^) or two asterisks (^∗∗^) indicate significant differences between control and treatment as determined by a one-way ANOVA test (*t*-Student, *P* < 0.01 and *P* < 0.001, respectively). **(C)** Protein accumulation pattern of barley C1A CysProt (HvPap-1, -19, -6 and -16) after 24 h of elicitor treatments, assayed by immunoblot. Proteins were extracted from 7 days old barley leaves without treatment (control) and treated with flagellin (Flg22) or chitosan (Chit). Rubisco protein expression was analyzed using a specific antibody against its large subunit.

For elicitor treatments, flg22, a 22-amino acid sequence of the conserved N-terminal part of bacterial flagellin, was used. This peptide is known to activate plant defense mechanisms. The effect of chitosan, which is a structural element in the exoskeleton of crustaceans and cell walls of fungi, on protease expression was also tested. A molecular and biochemical characterization of selected C1A CysProt members was performed including mRNA quantification (**Figures 1A**, **B**) and protein accumulation (**Figure 1C**) in response to elicitor treatments. The results, expressed as mRNA levels normalized to the constitutively active barley *cyclophilin* gene, revealed that after 24 h of flg22 treatment the cathepsin F, *HvPap-1* gene, was significantly induced in treated leaf samples (**Figure 1A**). After 24 h of chitosan treatment the most abundant transcripts were *HvPap-1* and *HvPap-19*, encoding cathepsin F- and B-like, respectively (**Figure 1B**). Besides, differences in expression were detected after flg22 treatment in the case of *HvPap-16* and after chitosan treatment for *HvPap-6*, both proteases belonging to cathepsin-L group. These results were companied by immunoblot assays using antibodies against specific peptides of HvPap-1, HvPap-6, HvPap-16 and HvPap-19 CysProt (**Figure 1C**). In some cases the results point out a direct correlation between transcript and protein accumulation patterns, as for HvPap-1 after chitosan treatment. Bands increased their signal in HvPap-19 not only after chitosan treatment but also after flg22 elicitation. A weak induction was observed for the cathepsin L-like proteins HvPap-6 and -16 in chitosan treated plants, while no remarkable differences were detected for flg22 treatments. Elicitors did not alter the amount of Rubisco in barley leaves.

### Expression of C1A Proteases Is Altered in Barley Leaves during the Response to *M. oryzae* and *T. urticae*

**FIGURE 2:**
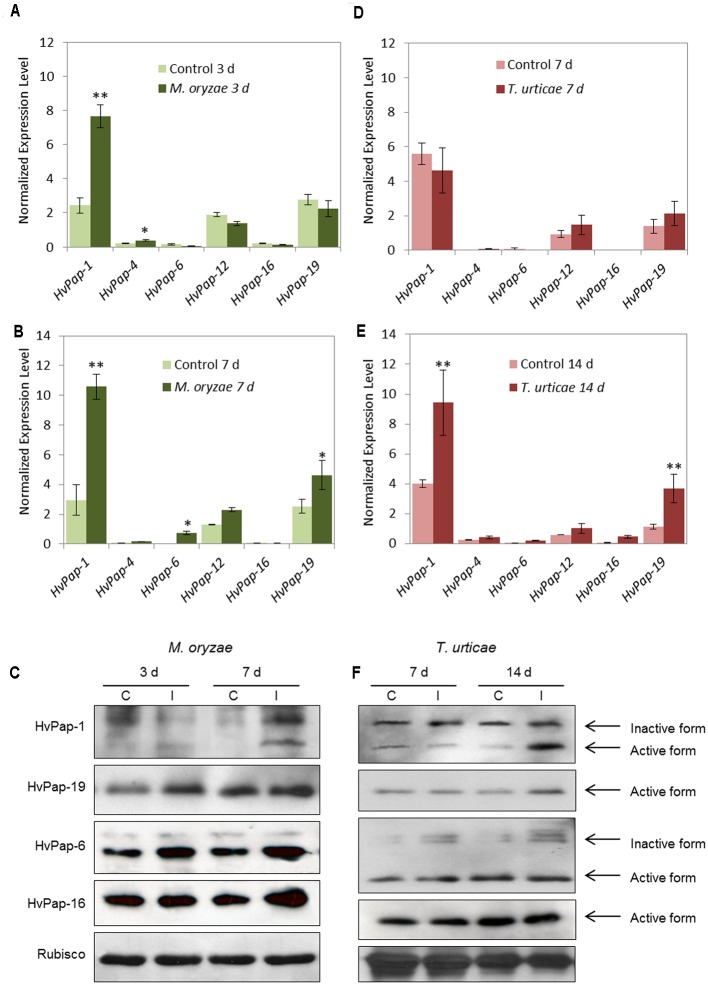
mRNA expression levels of barley C1A CysProt genes after *M. oryzae* infection and *T. urticae* infestation. Expression of barley CysProt genes (*HvPap-1, -4, -6, -12, -16* and *-19*) in *M. oryzae* infected (dark green) and non-infected (light green) barley leaves at **(A)** 3 days and **(B)** 7 days of treatment, and in *T. urticae* infested (dark red) and non-infested (light red) barley leaves at **(D)** 7 days and **(E)** 14 days of treatment. Data were determined by RT-qPCR and expressed as mRNA levels of C1A CysProt genes normalized to barley *cyclophilin* mRNA content. One (^∗^) or two asterisks (^∗∗^) indicate significant differences between control and treatment as determined by a one-way ANOVA test (*t*-Student, *P* < 0.01 and *P* < 0.001, respectively). Protein accumulation patterns of barley C1A CysProt (HvPap-1, -19, -6 and -16) after **(C)** *M. oryzae* infection and **(F)** *T. urticae* infestation assayed by immunoblot. Proteins were extracted from barley leaves at 3, 7, and 14 d of infection/infestation (I), and non-infection/infestation (C) treatments. Bands corresponding to inactive and active forms of CysProt are indicated by arrows. Rubisco protein expression was analyzed using a specific antibody against its large subunit.

Since both elicitors induced the expression of some C1A CysProt, the next approach was to evaluate the effects of a pathogen, the fungus *M. oryzae*, and an herbivore, the spider mite *T. urticae*, in treated barley leaves. Firstly, the phenotype of damage was observed and the disease rating and leaf damage produced in the plant was recorded by scanning leaves at different days after infection/infestation (Supplementary Figure 1). The same CysProt studied after elicitor treatments were analyzed under *M. oryzae* and *T. urticae* attack. Results showed that at 3 dpi of *M. oryzae* the cathepsin F-like *HvPap-1* was up-regulated (**Figure 2A**). After 7 days of *M. oryzae* infection, *HvPap-1* maintained the highest mRNA levels; meanwhile significant overexpression of other protease genes such as *HvPap-19* and *HvPap-6*, cathepsins B- and L-like respectively, was also observed (**Figure 2B**). The *M. oryzae* infected and non-infected barley leaves were also analyzed by immunoblotting in order to detect whether the CysProt accumulated after 3 and 7 days of fungus infection (**Figure 2C**). As previously reported Velasco-Arroyo et al. (2016), the protein profile of some CysProt showed two bands of different size corresponding to the immature protein and the mature processed form (without the pro-peptide). Both bands of HvPap-1 increased their signal in damaged leaf samples at 7 days of *M. oryzae* infection. A similar induction pattern was observed for the active form of the HvPap-6 and slightly for the -16 cathepsin L-like proteins, which were accumulated after fungus infection, particularly at 7 days. In addition, an induction pattern was observed for the active form of the cathepsin B-like HvPap-19, which was already accumulated at 3 days of treatment. No differences were detected for Rubisco levels in barley leaves.

On the other hand, at 7 days of *T. urticae* infestation (**Figure 2D**), no appreciable differences were shown in the expression of selected C1A CysProt. However, at 14 d of mite attack, the CysProt genes *HvPap-1* and *HvPap-19* were significantly up-regulated in infested leaves (**Figure 2E**). The protein profiles of CysProt HvPap-1, HvPap-6, HvPap-16 and HvPap-19 were analyzed by immunoblotting assays using total protein from *T. urticae* infested and non-infested barley leaves (**Figure 2F**). The lower (active mature protein) and the higher (inactive form) bands of these CysProt were observed in HvPap-1 and -6. HvPap-1 active form strongly increased after 14 days of mite infestation, whilst HvPap-6 revealed an increment of the inactive form after infestation in both time points, 7 and 14 days. HvPap-19 active form increased at 14 days of mite attack, while no differences of level of HvPap-16 were observed, in control or infested leaves at the two observed time points. Besides, 7 days assayed barley leaves showed a higher quantity of Rubisco protein than 14 days plants. And at this time, a slightly reduction of Rubisco in infested leaves could be appreciated.

### Transgenic Barley *HvPap-1* Lines Show Phenotypical Differences after *M. oryzae* Infection

**FIGURE 3:**
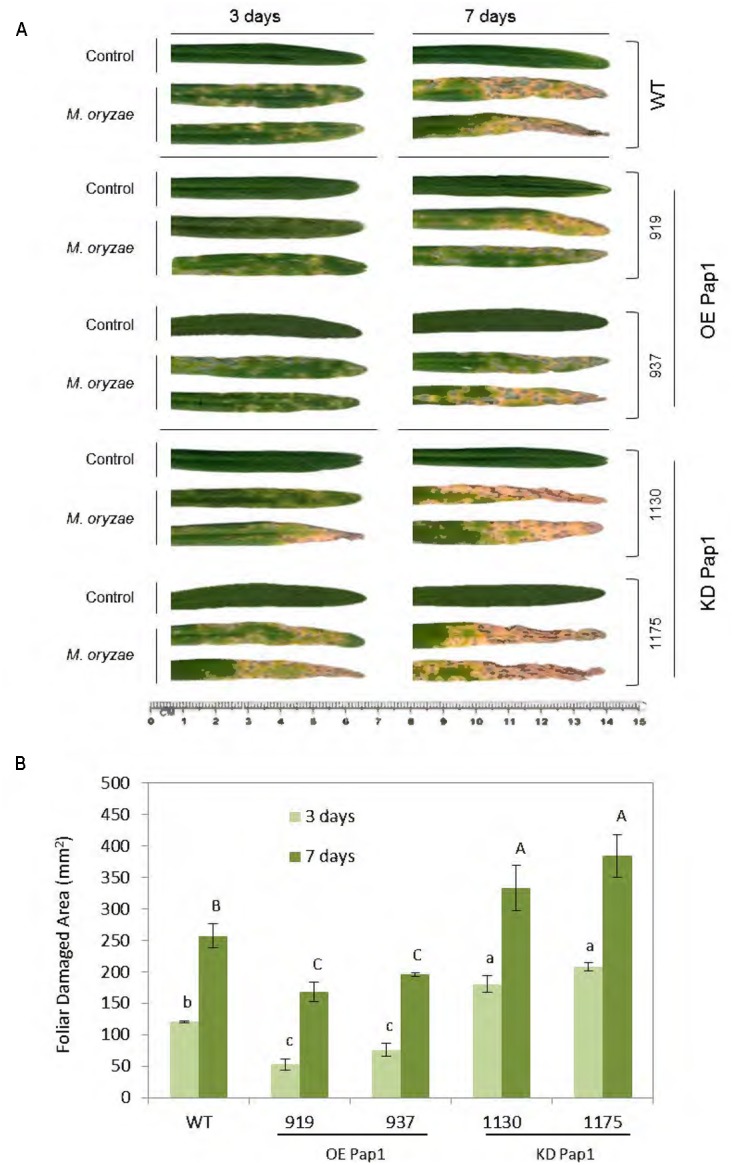
Images of the oldest leaf of transgenic and wild-type barley lines during *M. oryzae* attack, along 3 and 7 days of infection treatment or non-infection as control. **(A)** Leaves from *HvPap-1* overexpressing lines (OE Pap1, 919 and 937), silencing lines (KD Pap1, 1130 and 1175) and wild-type (WT) plants. **(B)** Quantification of leaf damage on barley transformed (OE Pap1and KD Pap1) and non-transformed (WT) plants, after 3 (light green) and 7 (dark green) d of *M. oryzae* infection. Damage was measured as mm^2^ of injured foliar area and is represented as mean ± SE of seven old leaves measurements from seven independent plants per treatment. Different letters indicate significant differences (*P* < 0.01, one-way ANOVA Student Newman–Keuls test).

HvPap-1 CysProt may have a relevant role in plant responses to biotic stresses, since it was the most up-regulated protease after all tested biotic treatments. Therefore, we used homozygous barley plants overexpressing or silencing the *HvPap-1* gene to carry out *in vivo* experiments to test the resistance or susceptibility of modified plants toward the selected barley’s pathogen and herbivore. The implication of the HvPap-1 CysProt in the response to *M. oryzae* was first analyzed by comparing transgenic and non-transgenic plants after fungus infection (Supplementary Figure 2A). As expected, leaves from all infected plants presented more damage than those grown under control conditions (**Figure 3A**). After 3 days of infection, leaves from *HvPap-1* knock-down lines (KD Pap1, 1130 and 1175) showed more damage than *HvPap-1* overexpression lines (OE Pap1, 919 and 937) or wild-type (WT) barley plants. After 7 days of *M. oryzae* attack, the highest susceptibility of KD Pap1 plants was apparent, whilst the OE Pap1 lines presented fewer symptoms of damage and seemed less susceptible to the fungus than WT plants. These observations were corroborated by analyzing the leaf area damaged after infection and by comparing total damaged area in WT and transgenic infected lines (**Figure 3B**). The WT plants showed significantly lower damaged foliar area than KD Pap1 plants and greater damage than OE Pap1 transgenic lines at both time points, 3 and 7 days.

To study the effect of the transgenic barley plants on the fungus performance the presence of *M. oryzae* was measured by quantifying mRNA levels corresponding to the small subunit of ribosomal RNA (*Mo28S-rRNA*) (Supplementary Figure 3A). The results showed that at 5 days of infection a significantly higher quantity of fungus mRNA was appreciated in KD Pap1 barley plants indicating that these plants seem more susceptible to *M. oryzae* in comparison to WT and OE Pap1 plants. Finally, all lines showed increased levels of fungus mRNA at 7 d of infection, although the OE Pap1 lines presented the lowest levels, remaining as the less susceptible to the attack.

### Transgenic Barley *HvPap-1* Lines Show Phenotypical Differences after *T. urticae* Infestation

**FIGURE 4:**
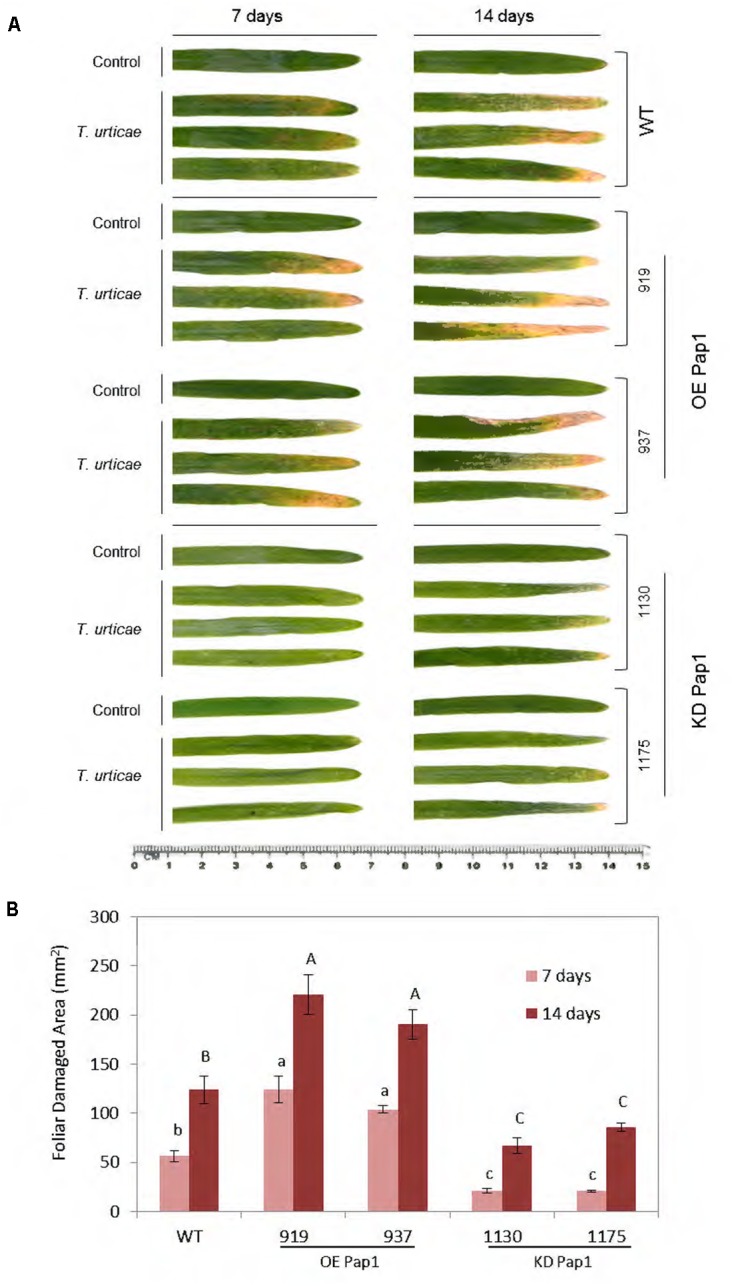
Images of the oldest leaf of transgenic and wild-type barley lines during *T. urticae* attack, along 7 and 14 days of infestation or non-infestation as control. **(A)** Leaves from *HvPap-1* overexpressing lines (OE Pap1, 919 and 937), silencing lines (KD Pap1, 1130 and 1175) and wild-type (WT) plants. **(B)** Quantification of leaf damage on barley transformed (OE Pap1 and KD Pap1) and non-transformed (WT) plants, after 7 (light red) and 14 (dark red) d of *T. urticae* infestation. Damage was measured as mm^2^ of injured foliar area and is represented as mean ± SE of seven old leaves measurements from seven independent plants per treatment. Different letters indicate significant differences (*P* < 0.01, one-way ANOVA Student Newman–Keuls SNK test).

To compare plant responses against a pathogen and an herbivore, WT and homozygous plants overexpressing or silencing the CysProt HvPap-1 were used to carry out *in vivo* experiments to test the susceptibility of modified plants upon *T. urticae* infestation (Supplementary Figure 2B). The participation of HvPap-1 in the response to *T. urticae* was first analyzed by comparing transgenic and non-transgenic plants after mite infestation (**Figure 4**). As expected, leaves from infested plants were more affected than those grown under non-infested conditions (**Figure 4A**). After 7 days of infection, the most damaged leaves were observed in the OE Pap1 (919 and 937) lines, followed by the WT, whereas the KD Pap1 (1130 and 1175) lines presented only slight damages. At 14 days of mite infestation the phenotypic observations were further manifested, the differences among lines were stronger, OE Pap1 lines were much more injured than KD Pap1 lines, and WT plants showed an intermediate damaged phenotype. Furthermore, spider mite feeding effects were analyzed by quantifying the injured leaf area (**Figure 4B**). These results corroborated the phenotypical observations. The OE Pap1 lines had significant larger damaged foliar area than WT plants, and the KD Pap1 lines showed significant lesser injury than WT plants.

To study whether the transgenic barley plants affected *T. urticae* performance, the presence of the mite at different time points of infestation was analyzed by quantifying *T. urticae* Ribosomal Protein 49 (*TuRP49*) mRNA levels (Supplementary Figure 3B). The results showed a noticeable increment of mite mRNA in the OE Pap1 lines followed by WT barley plants at 7 days of infestation. At 14 days of *T. urticae* feeding, all lines showed increased levels of mite mRNA, although the KD Pap1 lines presented the lowest levels.

### Transgenic Barley *HvPap-1* Lines Show Alteration in Other CysProt after *M. oryzae* and *T. urticae* Treatments

**FIGURE 5:**
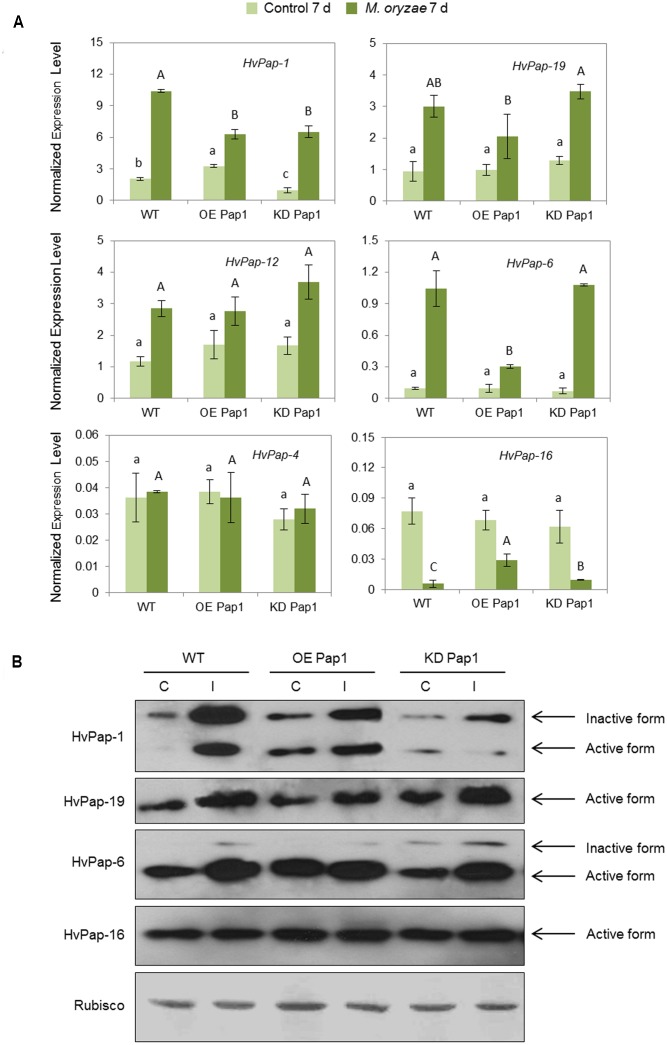
Messenger expression levels of C1A CysProt genes (*HvPap-1, -19, -12, -6, -4* and -*16*) in transgenic *HvPap-1* overexpressing (OE Pap1, 919) and silencing (KD Pap, 1175) lines, and wild-type (WT) barley plants during *M. oryzae* infection, assayed by RT-qPCR. **(A)** Total RNA was extracted from leaves after 7 d of infection (dark green) and non-infected leaves (light green). Data were expressed as mRNA levels of C1A CysProt genes normalized to barley *cyclophilin* mRNA content. Different letters indicate significant differences (*P* < 0.01, one-way ANOVA Student Newman-Keuls test). **(B)** Protein accumulation patterns of C1A CysProt (HvPap-1, -19, -6 and -16) in transgenic (OE Pap1 and KD Pap1) and wild-type (WT) barley plants during *M. oryzae* infection assayed by immunoblot. Total protein was extracted form leaves after 7 days of infection (I) and non-infected leaves (C). Rubisco protein expression was assayed using a specific antibody against its large subunit.

The expression patterns of several CysProt were investigated in control and treated plants, either by *M. oryzae* or *T. urticae*, in WT and transformed lines. As shown in **Figure 5A** and Supplementary Figure 4, *HvPap-1* transcripts increased in plants infected with *M. oryzae* independently of the transgene insertion, although transcript levels were lower in transgenic lines than in WT. The mRNA profile of other genes encoding CysProt was also analyzed in these transgenic plants. *HvPap-19* and *HvPap-12* were up-regulated in infected WT, OE Pap1 and KD Pap1 lines. *HvPap-6* sharply increased in KD Pap1 and WT when compared to the controls without infection but it did moderately in OE Pap1. No differences among treatments or lines were found for *HvPap-4*, and the *HvPap-16* gene was repressed in response to *M. oryzae* infection. HvPap-1, HvPap-19, HvPap-6 and HvPap-16 proteases were detected by immunoblot in protein extracts from control and *M. oryzae* infected leaves of transformed and non-transformed plants using specific antibodies (**Figure 5B**). The HvPap-1 protein increased not only in the overexpressing OE Pap1 lines in comparison with the WT, but also after the *M. oryzae* infection treatments. In contrast, HvPap-1 active form diminished in KD Pap1 lines. A slight increase of HvPap-19 and HvPap-6 proteins was also observed in leaves grown under infection. No alterations in the HvPap-16 protein levels were detected in transgenic lines compared to WT plants or comparing among treatments. No differences were detected in Rubisco levels in barley leaves neither lines nor treatments.

**FIGURE 6:**
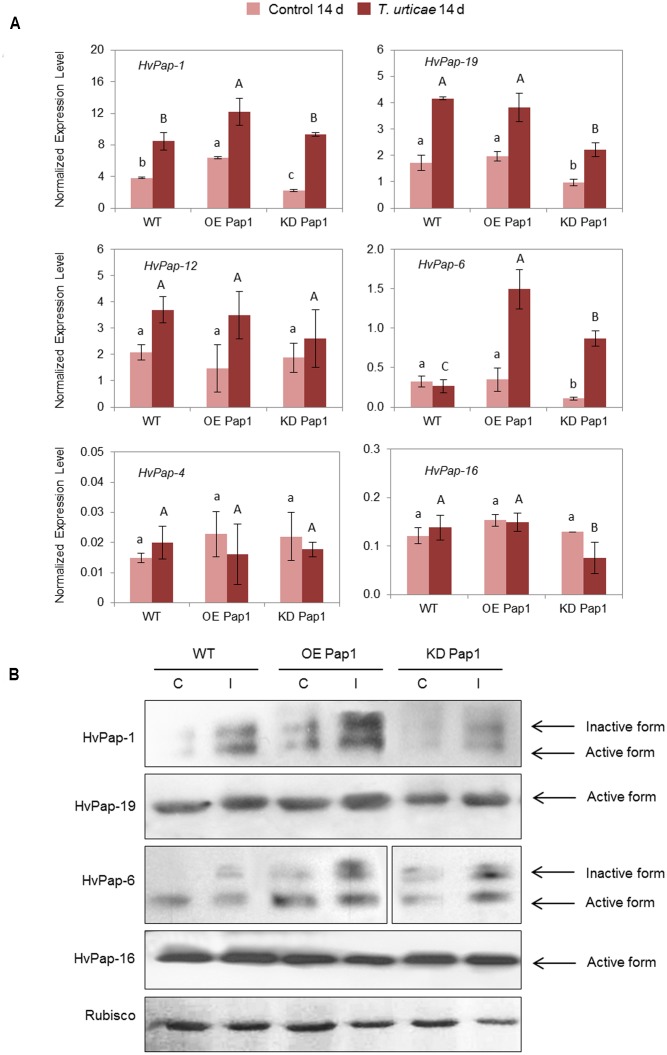
Messenger expression levels of C1A CysProt genes (*HvPap-1, -19, -12, -6, -4* and -*16*) in transgenic *HvPap-1* overexpressing (OE Pap1, 919) and silencing (KD Pap1, 1175) lines, and wild-type (WT) barley plants during *T. urticae* infestation, assayed by RT-qPCR. **(A)** Total RNA was extracted from leaves after 14 days of infestation (dark red) and non-infested leaves (light red). Data were expressed as mRNA levels of C1A CysProt genes normalized to barley *cyclophilin* mRNA content. Different letters indicate significant differences (*P* < 0.01, one-way ANOVA Student Newman–Keuls test). **(B)** Protein accumulation patterns of C1A CysProt (HvPap-1, -19, -6 and -16) in transgenic (OE Pap1 and KD Pap1) and wild-type (WT) barley plants during *T. urticae* infestation assayed by immunoblot. Total protein was extracted form leaves after 14 d of infestation (I) and non-infested leaves (C). Rubisco protein expression was assayed using a specific antibody against its large subunit.

As shown in **Figure 6A** and Supplementary Figure 5, *HvPap-1* transcripts increased in plants infested with *T. urticae* independently of the transgene insertion. The expression of the *HvPap-19* gene was up-regulated in infested WT, OE Pap1 and KD Pap1 lines, but KD Pap1 showed the lowest levels of this protease before and after infestation. *HvPap-12* was slightly induced after *T. urticae* attack and *HvPap-6* was highly induced in KD Pap1 and OE Pap1 lines. No remarkable differences were found for *HvPap-4* and *HvPap-16* genes. Immunoblotting assays were performed using protein extracts from control and *T. urticae* infested leaves (**Figure 6B** and Supplementary Figure 6). The inactive and active HvPap-1 protein forms increased not only in the overexpressing OE Pap1 lines in comparison with WT and KD Pap1, but also after the *T. urticae* infestation treatments in all lines. A slight increase of HvPap-19 and the inactive form of HvPap-6 was also observed in leaves grown under infestation treatments. No remarkable alterations in the HvPap-16 protein levels were detected. Low differences were detected in Rubisco levels, which were slightly reduced in all lines after mite treatment.

### Silencing and Overexpression of *HvPap-1* Affect the Expression of Other Barley Genes

**FIGURE 7:**
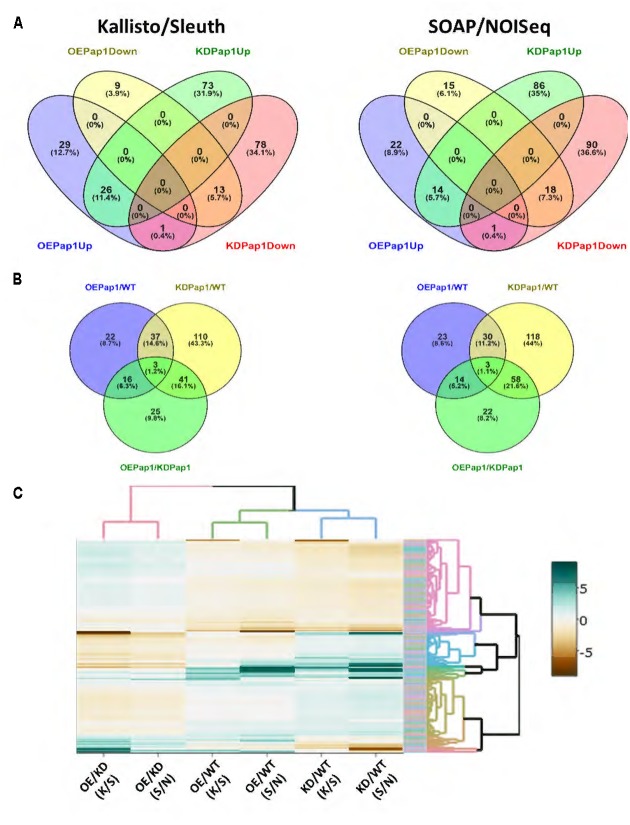
Differentially expressed genes (DEGs) in overexpressing (OE Pap1, 919) and silencing (KD Pap1, 1175) *HvPap-1* transgenic lines. **(A)** Venn diagrams showing up and down regulated genes in OE Pap1 and KD Pap1 transgenic plants. **(B)** Venn diagrams show shared DEGs between different genotype comparisons. **(C)** Heatmap showing the expression levels in the different genotypes of all the obtained DEGs by Kallisto/Sleuth (K/S) and SOAP/NOISeq (S/N) analyses.

To obtain more information on the molecular basis responsible to the differential and opposite responses of transgenic *HvPap-1* lines against *M. oryzae* and *T. urticae* stresses, a RNA-seq analysis was performed in 7 days old non-stressed plants. To test the robustness of the analysis, two different approaches were used to obtain DEGs between lines. Only a limited number of genes were detected as DEGs by either analytical methods, with around 70% of DEGs exclusively detected by the Kallisto/Sleuth or the SOAP/NOIseq methods (Supplementary Figure 7). When the expression of the transgenic lines was compared with the wild-type, only one DEG, *HvPap-1*, was up-regulated in overexpressing lines and down-regulated in silencing lines (**Figure 7A**). No other DEG was inversely regulated in both lines. On the contrary, a substantial number of DEGs were up or down-regulated when compared with the expression in wild type in both silencing and overexpressing lines. In the same way, only three DEGs were detected in the three OE Pap1/WT, KD Pap1/WT and OE Pap1/KD Pap1 analyses (**Figure 7B**) with around 60% of DEGs only detected in a unique comparison.

To further analyze the expression of the DEGs, a heatmap showing the differential values obtained by the two methods in all comparisons was performed. As expected, the results of both methods on the same comparison tend to show similar patterns (**Figure 7C**). Besides, clustering shows that only a reduced number of genes showed the OE > WT > KD or KD > WT > OE expression patterns. When we focused in the OE Pap1 *vs* KD Pap1 comparison, a robust relationship of DEGs with any specific biological process was not observed (Supplementary Table 3). Thus, the DEG lists were screened to detect genes potentially involved in the response to biotic stresses (Supplementary Tables 4, 5). From these analyses, several genes were selected as potential candidates to be involved in the different responses to biotic stresses in OE Pap1 and KD Pap1 plants. Two of these genes belong to the germin family, previously related to basal resistance against fungi (Zimmermann et al., 2006), and two genes were protease inhibitors, proteins broadly related to herbivore resistance (Schlüter et al., 2010), of the I12 Bowman–Birk and I13 Potato-I families. A comparative analysis of the expression data obtained in the RNA-seq experiment showed the highest expression of germin-like genes in the OE Pap1 line and the lowest in the KD Pap1 line (Supplementary Figure 8). On the contrary, the protease inhibitor belonging to the I13 family was more expressed in the KD Pap1 line and less expressed in the OE Pap1 line. Finally, the protease inhibitor from the I12 family exhibited lower expression in the OE Pap1 than in the KD Pap1 and WT plants.

### Germin-Like and Protease Inhibitors Respond Differentially to Biotic Stresses in *HvPap-1* Transgenic Barley Lines

**FIGURE 8:**
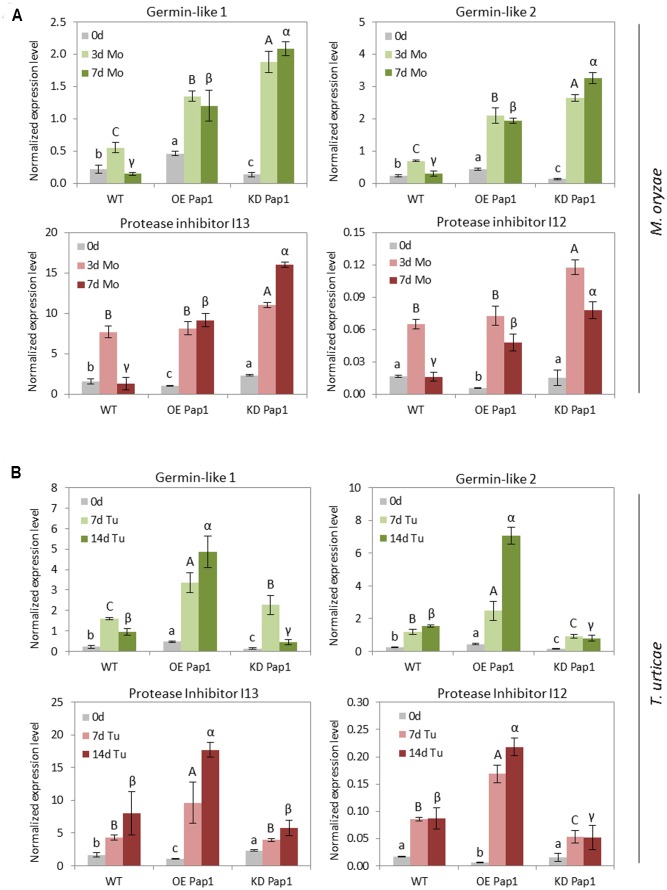
Messenger expression levels of germin-like genes and protease inhibitors genes from I12 Bowman-Birk and I13 Potato Inhibitor I families, in transgenic *HvPap-1* overexpressing (OE Pap1, 919) and silencing (KD Pap1, 1175) lines, and wild-type (WT) barley plants, assayed by RT-qPCR. Total RNA was extracted form leaves after **(A)** three (3d) or seven (7d) days of *M. oryzae* infection (light and dark green) or **(B)** seven (7d) or fourteen (14d) days of *T. urticae* infestation (light and dark red) and non-treated leaves (light gray). Data were expressed as mRNA levels normalized to barley *cyclophilin* mRNA content. Different letters indicate significant differences (*P* < 0.01, one-way ANOVA Student Newman–Keuls test).

The selected DEGs were analyzed by RT-qPCR in control or treated *HvPap-1* silencing and overexpression barley plants as well as in WT (**Figure 8** and Supplementary Figure 9). The variable expression patterns of these genes in non-treated control plants validate the data obtained by the RNA-seq analysis, showing the highest expression of germin-like genes in the OE Pap1 line and the lowest in the KD Pap1 line, whilst the protease inhibitors genes were less expressed in the OE Pap1 line. After biotic stress treatments, germin-like and protease inhibitor genes increased their levels of expression in all lines but with a different pattern depending on the biotic treatment and the plant genotype (**Figure 8**). Germin-like genes were remarkably more induced by *M. oryzae* in the KD Pap1 plants than in WT or OE Pap1 plants. Conversely, the induction of germin-like genes was greater in OE Pap1 plants after 14 days of *T. urticae* infestation. Protease inhibitor genes followed a similar induction pattern after 3 days of *M. oryzae* infection in all genotypes, but their expression showed a differentially reduced pattern in WT plants after 7 days of infection. In response to spider mite infestation, protease inhibitor genes were considerably more induced in OE Pap1 than in WT or KD Pap1 plants.

## Discussion

Recent evidences (reviewed by Misas-Villamil et al., 2016) support the role of C1A papain-like proteases in plant immunity. Silencing of plant C1A CysProt leads to a higher susceptibility against specific pathogens and herbivores, and several papain-like proteases have been described as targets of pathogen effectors. As several C1A proteases are induced both during senescence and in response to biotic stimuli (Pechan et al., 2000; Kempema et al., 2015), we wonder if members of the well-characterized barley C1A family, previously related to abiotic stresses (Diaz-Mendoza et al., 2014; Velasco-Arroyo et al., 2016), had also a role in plant defense. As a first approach, a leaf treatment with the molecules flagellin and chitosan, known to elicit defense response in plants (Newman et al., 2013), allowed us to identify several barley C1A CysProt induced after these elicitors treatments. Among them, the cathepsin F -like *HvPap-1* showed the highest levels after the treatments.

Most C1A proteases have been associated to the response toward a unique pathogen/herbivore. Therefore, there is scarce information on the spectrum of organisms that trigger a defensive response involving a particular protease. The best described example corresponds to the pair Rcr3/Pip1 proteases of tomato, which functionally diverged after duplication. Whereas *rcr3* null mutants were more susceptible to the fungus *Cladosporium fulvum*, the nematode *Globodera rostochiensis* and the oomycete *P. infestans*, those lines depleted for the Pip1 protease were more susceptible to *C. fulvum*, *P. infestans* and the bacteria *Pseudomonas syringae* (Lozano-Torres et al., 2012; Ilyas et al., 2015). Besides, the maize Mir1 protein conferred enhanced resistance to different herbivores, including caterpillars, root-feeding herbivores and aphids (Pechan et al., 2000, 2002; Gill et al., 2011; Louis et al., 2015). However, any study has been focused in the ability of a C1A protease to participate in the defense against both pathogens and herbivores. Thus, the next step was to characterize the transcriptional responses of barley C1A CysProt under a pathogen and an herbivore treatment. The expected result was the induction of some proteases, since there are numerous examples of C1A CysProt induced by phytophagous attack (Shindo and van der Hoorn, 2008). The infection with the fungus *M. oryzae* and the infestation with the mite *T. urticae* confirmed this hypothesis and corroborated the putative defensive role of the response of barley C1A CysProt to elicitors. Several barley CysProt were induced by both stresses and, again, *HvPap-1* gene presented the greatest induction at the transcriptional level.

Previously characterized homozygous transgenic barley lines silencing and overexpressing the *HvPap-1* encoding gene (Diaz-Mendoza et al., 2016), allowed us to elucidate the *in vivo* participation of this protease during biotic stresses. If the role of HvPap-1 was directly related to plant resistance, the expected result of silencing its gene expression would be a higher susceptibility to pathogen/herbivore treatments. On the contrary, the overexpression of this gene would lead to greater resistance to biotic stresses. In agreement with this hypothesis, *HvPap-1* silencing barley lines showed a higher susceptibility to *M. oryzae* infection, which is in line with the increased susceptibility to *P. infestans* in tomato and potato plants silencing the CysProt C14 (Kaschani et al., 2010; Bozkurt et al., 2011), the enhanced susceptibility to *Erysiphe cruciferarum* and *B. cinerea* in knock-out plants for the RD21 or AtCEP1 CysProt in *Arabidopsis* (Shindo et al., 2012; Höwing et al., 2014), or the further growth of bacterial pathogens observed in *Nicotiana benthamiana* plants silencing the *NbCathB* gene (Gilroy et al., 2007). Likewise, barley *HvPap-1* overexpressing plants remained less susceptible to *M. oryzae* when compared to WT plants; similarly to the improved resistance to the fungal pathogen *B. cinerea* achieved through the overexpression in *Arabidopsis* of the *AcCP2* gene encoding a C1A CysProt from pineapple fruit (Wang et al., 2014). Surprisingly, despite a common induction after biotic stress, findings revealed a very different role of genetically manipulated plants in defense against the pathogen *M. oryzae* and the herbivore *T. urticae*. After spider mite infestation, *HvPap-1* silencing barley plants remain less susceptible to the mite attack, while the *HvPap-1* overexpression lines showed the greatest damage. This finding represents the opposite trend to that described in previous reports concerning a higher deleterious effect on phytophagous herbivores fed on leaves containing papain or in maize callus overexpressing the CysProt Mir1 (Pechan et al., 2000, 2002; Konno et al., 2004). This antagonistic behavior should be related to the defensive mechanism affected by HvPap-1. However, little is known on how protease activity triggers defensive responses. *Arabidopsis* cathepsin B-like CysProt have been associated to the hypersensitive response against bacterial phytopathogens (Gilroy et al., 2007; McLellan et al., 2009), probably due to their proteolytic activity, since they have also been involved in programmed cell death induced by abiotic stresses (Ge et al., 2016). Likewise, apoplastic maize C1A CysProt are necessary to induce the transcription of defense-related genes upon *Ustilago maydis* infection (van der Linde et al., 2012a,b), and their catalytic activity is needed to trigger cell death after treatment with the wound-inducible compound 10-OPEA (Christensen et al., 2015). Despite this scarce information, some cues can be extracted from our experiments. The timing of induction in response to the biotic stress is organism-specific. Whereas *HvPap-1* induction was clearly observed after 3 days of *M. oryzae* infection, the induction was only reported after 14 days of *T. urticae* infestation. This could imply different signaling pathways involved in the induction of *HvPap-1*. After *M. oryzae* infection, a quick induction of *HvPap-1* could be related to a direct implication in the signaling cascade leading to a defensive response to the fungus. The retarded induction of *HvPap-1* after spider mite infestation could be more related to senescence processes associated to leaf damage. This hypothesis is supported by the fact that jasmonic acid not only triggers defensive responses against herbivores but also leaf senescence (Reinbothe et al., 2009), and the reported induction of the jasmonic acid biosynthesis and signaling pathways after spider mite feeding (Ament et al., 2004; Martel et al., 2015; Santamaria et al., 2017). Therefore, if the induction of *HvPap-1* is due to different mechanisms, the analysis of the transcriptome in silencing or overexpressing *HvPap-1* lines could help to understand the differential responses to pathogen and herbivore stresses. Expression analyses on C1A CysProt point out to differential regulation of gene expression in these lines. The expression levels of several CysProt, such as *HvPap-6* and *-19*, were significantly different before and/or after infestation between OE Pap1 and KD Pap1 plants. To achieve more robust results, two different methods were used to analyze the transcriptomic data and to obtain differentially expressed genes. From these analyses, any known biological process was found to be clearly affected, but some genes related to pathogen/herbivore defense were identified. Interestingly, two genes belonging to the germin family were overexpressed in OE Pap1 plants and two protease inhibitors were repressed in KD Pap1 plants. Germin-like proteins have been related to basal resistance against the fungus *Blumeria graminis* in barley (Zimmermann et al., 2006). Besides, a germin-like protein, OsGLP2-1 overexpressed in rice conferred resistance to the blast fungus *M. oryzae* probably due to the accumulation of H_2_O_2_ and the activation of the JA-dependent pathway (Liu et al., 2016). In contrast, barley protease inhibitors overexpressed in *Arabidopsis* conferred resistance to *T. urticae* (Santamaria et al., 2012), and members of the I3 and I13 families are induced upon spider mite feeding in tomato (Martel et al., 2015). Thus, the transcriptional behavior of these genes correlates with the differential susceptibility against pathogens and herbivores showed by the genetically modified plants. Strikingly, the four genes were induced after both *M. oryzae* and *T. urticae* stresses. However, the highest induction of germin-like genes was observed in the most susceptible genotype to the pathogen, KD Pap1, and the highest induction of protease inhibitors was shown in OE Pap1, the most susceptible genotype to spider mites. These results, along to the observed lowest induction of *HvPap-1* in OE Pap1 lines after *M. oryzae* infection, would be in accordance with a scenario in which the less constitutively protected genotype needs to further induce an accumulation of proteins potentially required to resist the biotic attack.

## Conclusion

The results demonstrate the role of C1A CysProt in plant defense. However, caution should be taken when a gene is overexpressed in response to a biotic stress. The unexpected effects of the overexpression and silencing of an herbivore-induced CysProt highlight the importance of considering the interactions between different signaling pathways. This network implies not only components directly related to defense, but also to physiological processes such as leaf senescence. As different networks would involve different signaling pathways, the combination of final products will be the responsible of the organism-specific higher/lesser susceptibility caused by the genetic modification of the proteolytic machinery.

## Author Contributions

ID and MM conceived the research. MD-M performed most of the experimental research. BV-A collaborated in the biochemical and molecular analyses. MES participated in the mite-barley assays. MD-M, ID, and MM, participated in the design, the acquisition, analysis, or interpretation of data for the work. All authors contributed to final version of the manuscript.

## Conflict of Interest Statement

The authors declare that the research was conducted in the absence of any commercial or financial relationships that could be construed as a potential conflict of interest.

## Footnotes

1 http://pgsb.helmholtz-muenchen.de/plant/barley/index.jsp

2 http://bioinfogp.cnb.csic.es/tools/venny/index.html

3 https://github.com/yonicd/shinyHeatmaply

4 http://bioconductor.org/packages/release/bioc/html/topGO.html
