# Supplementary material for: HvPap-1 C1A Protease Participates Differentially in the Barley Response to a Pathogen and an Herbivore
Source: Front Plant Sci. 2017 Sep 12;8:1585. doi: 10.3389/fpls.2017.01585 (PMC5601043; doi:10.3389/fpls.2017.01585)
Supplement: Supplementary file 1 [file Data_Sheet_1.PDF]

Supplementary Material

HvPap-1 C1A protease participates differentially in the barley response to a pathogen and an herbivore

Mercedes Diaz-Mendoza, Blanca Velasco-Arroyo, M. Estrella Santamaria, Isabel Diaz, Manuel Martinez*

*** Correspondence:** Corresponding Author: m.martinez@upm.es

1 Supplementary Figures and Tables

1.1 Supplementary Figures

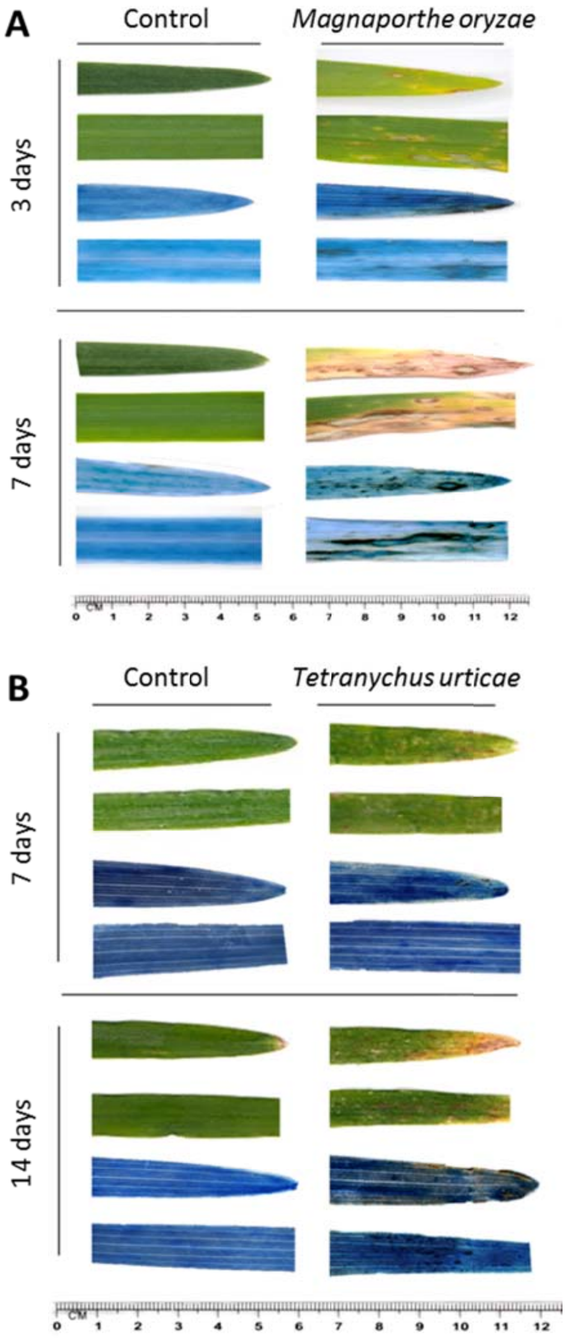

Supplementary Figure 1. Images of the apical and intermediate parts of the oldest leaf of barley plants after **(A)** 3 d and 7 d of *M. oryzae* infection and **(B)** 7 d and 14 d of *T. urticae* infestation, compared to controls without infection/infestation. In blue color, Trypan blue staining showing cell dead to highlight the damaged area.

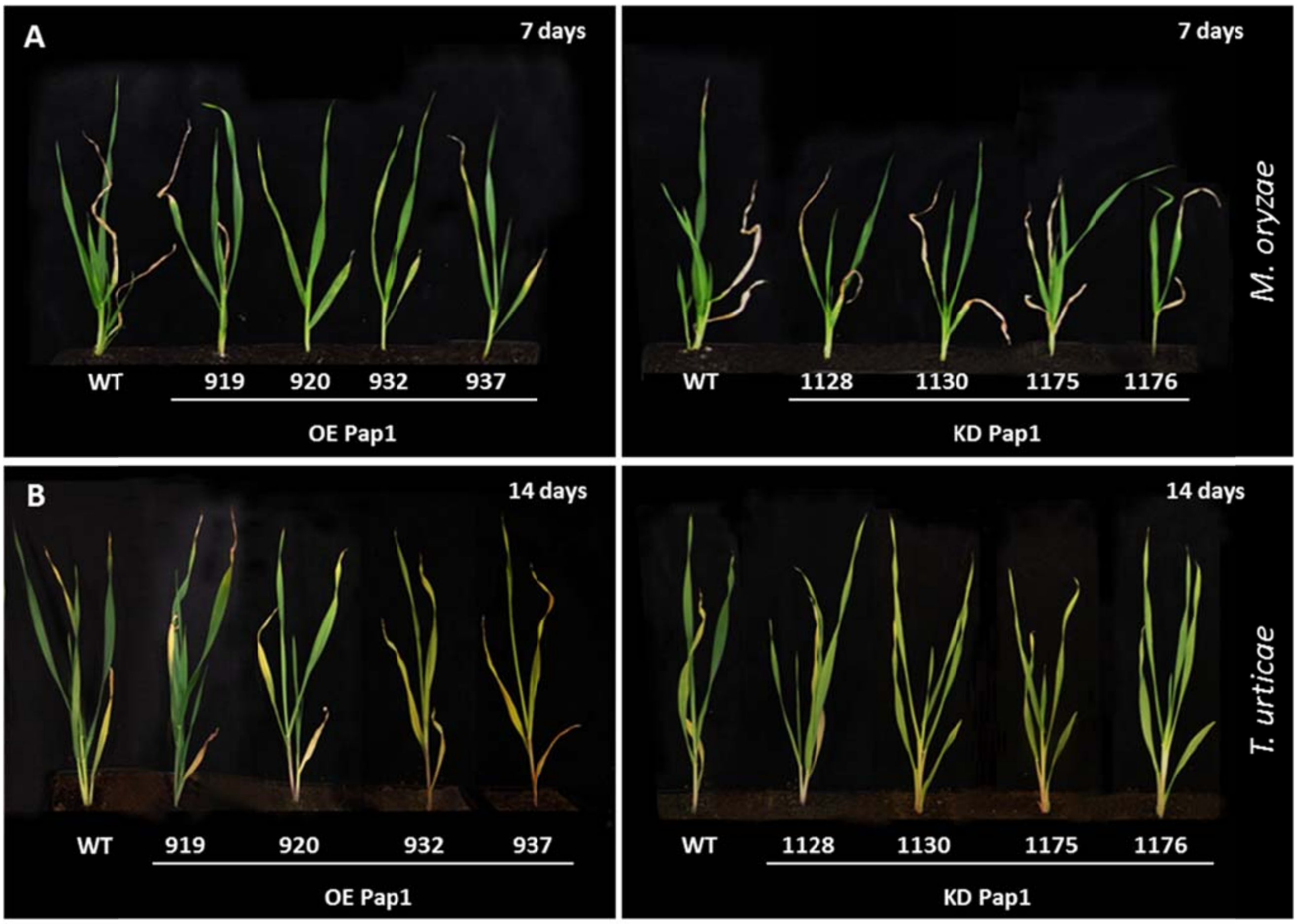

Supplementary Figure 2. Images of the whole barley plant after **(A)** 7 d of *M. oryzae* infection and **(B)** 14 d of *T. urticae* infestation from wild-type (WT), *HvPap-1* overexpressing lines (OE Pap1 919, 920, 932 and 937) and *HvPap-1* silencing lines (KD Pap1 1128, 1130, 1175 and 1176).

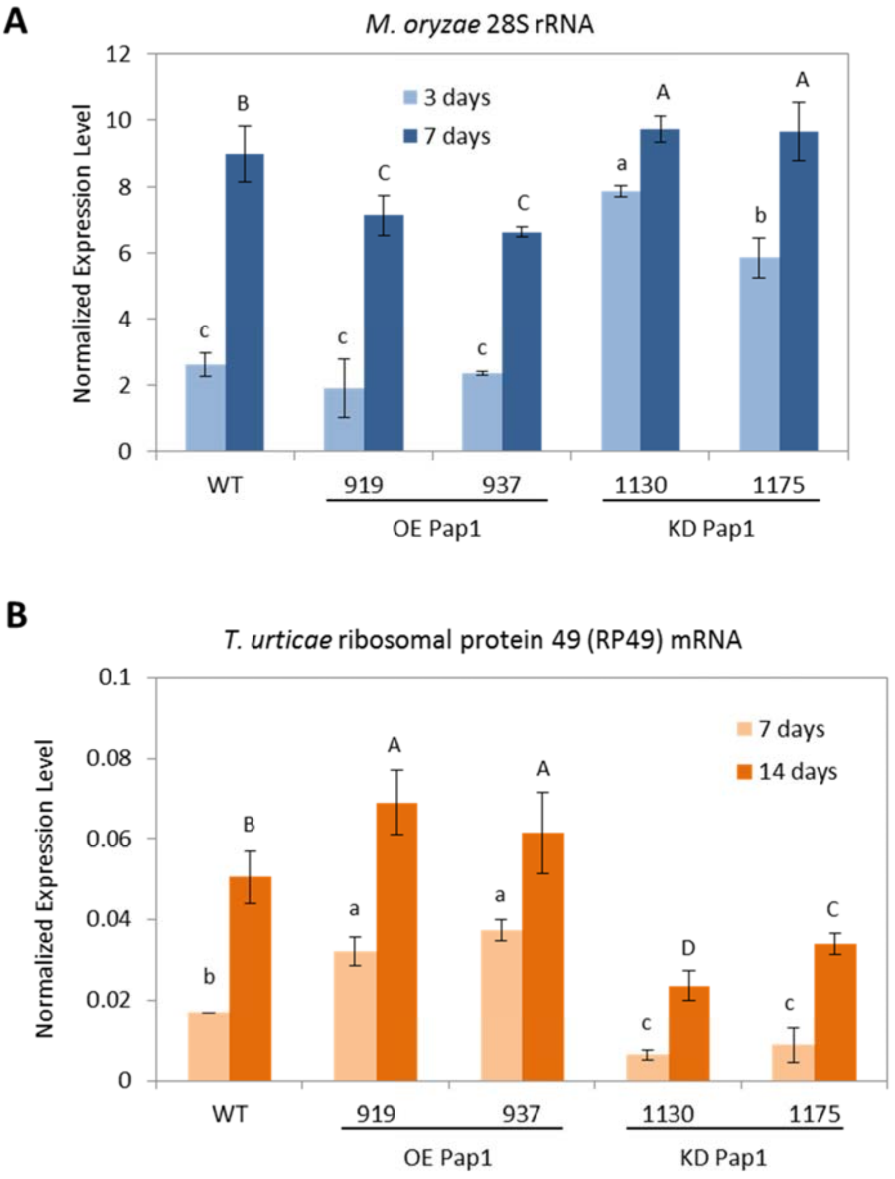

Supplementary Figure 3. Effect of transgenic barley lines overexpressing (OE Pap 919 and 937) or silencing (KD Pap1 1130 and 1175) the *HvPap-1* gene, and wild-type (WT) plants on *M. oryzae* and *T. urticae* performance. **(A)** Quantification of *M. oryzae* small subunit of ribosomal RNA (*Mo28S-rRNA*) mRNA expression levels, at 3 (light blue) and 7 (dark blue) d after *M. oryzae* infection. **(B)** Quantification of *T. urticae* Ribosomal Protein 49 (*TuRP49*) mRNA expression levels, at 7 (light orange) and 14 (dark orange) d after *T. urticae* infestation. Different letters indicate significant differences (P<0.01, one-way ANOVA Student Newman-Keuls test).

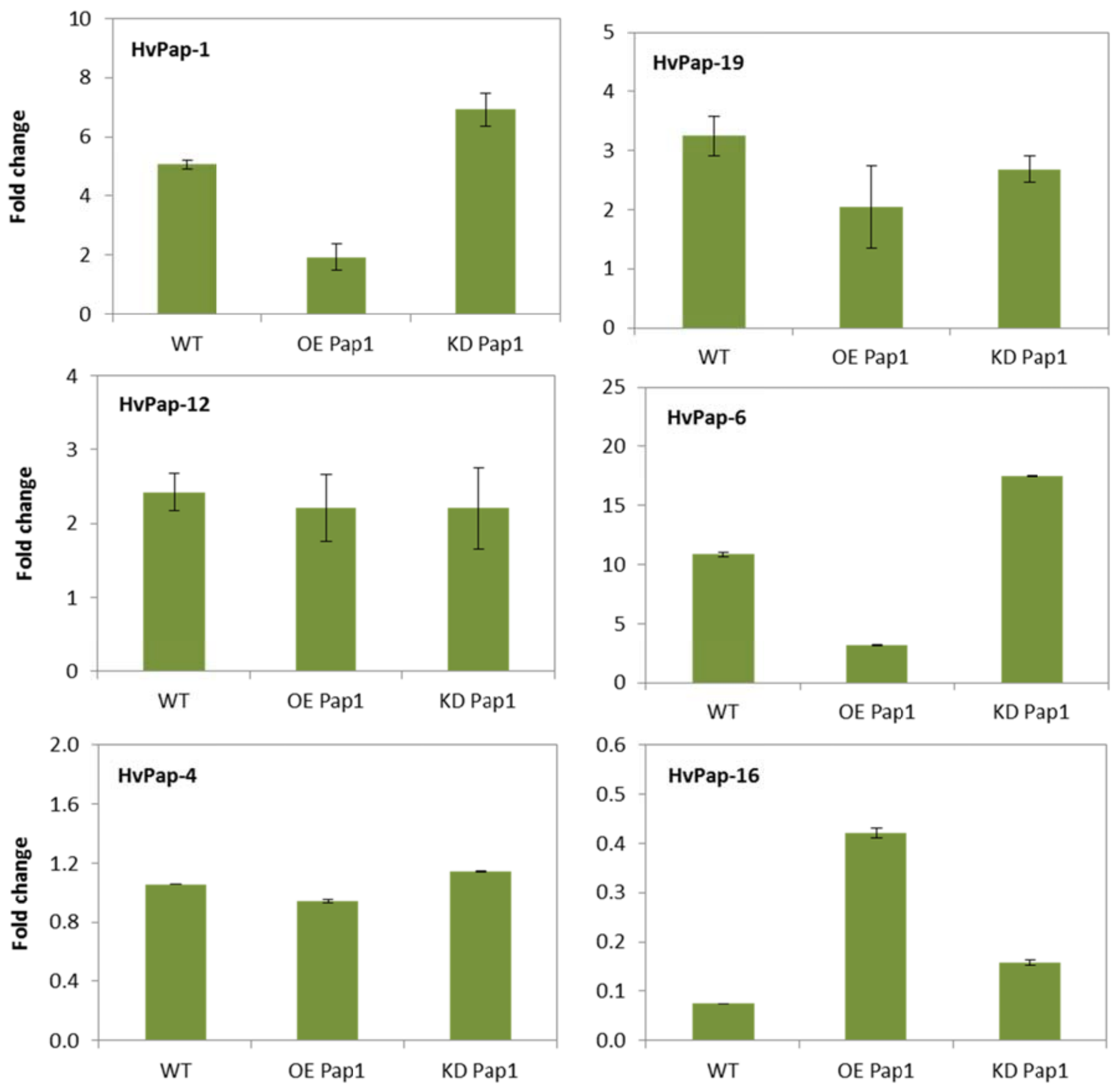

Supplementary Figure 4. Fold change in the expression of C1A CysProt genes (*HvPap-1, -19, -12, -6, -4* and *-16*) in transgenic *HvPap-1* overexpressing (OE Pap1, 919) and silencing (KD Pap, 1175) lines, and wild-type (WT) barley plants after 7 d of *M. oryzae* infection, assayed by RT-qPCR. Data were expressed as relative expression to the mRNA levels of C1A CysProt genes in non-treated plants normalized to barley *cyclophilin* mRNA content.

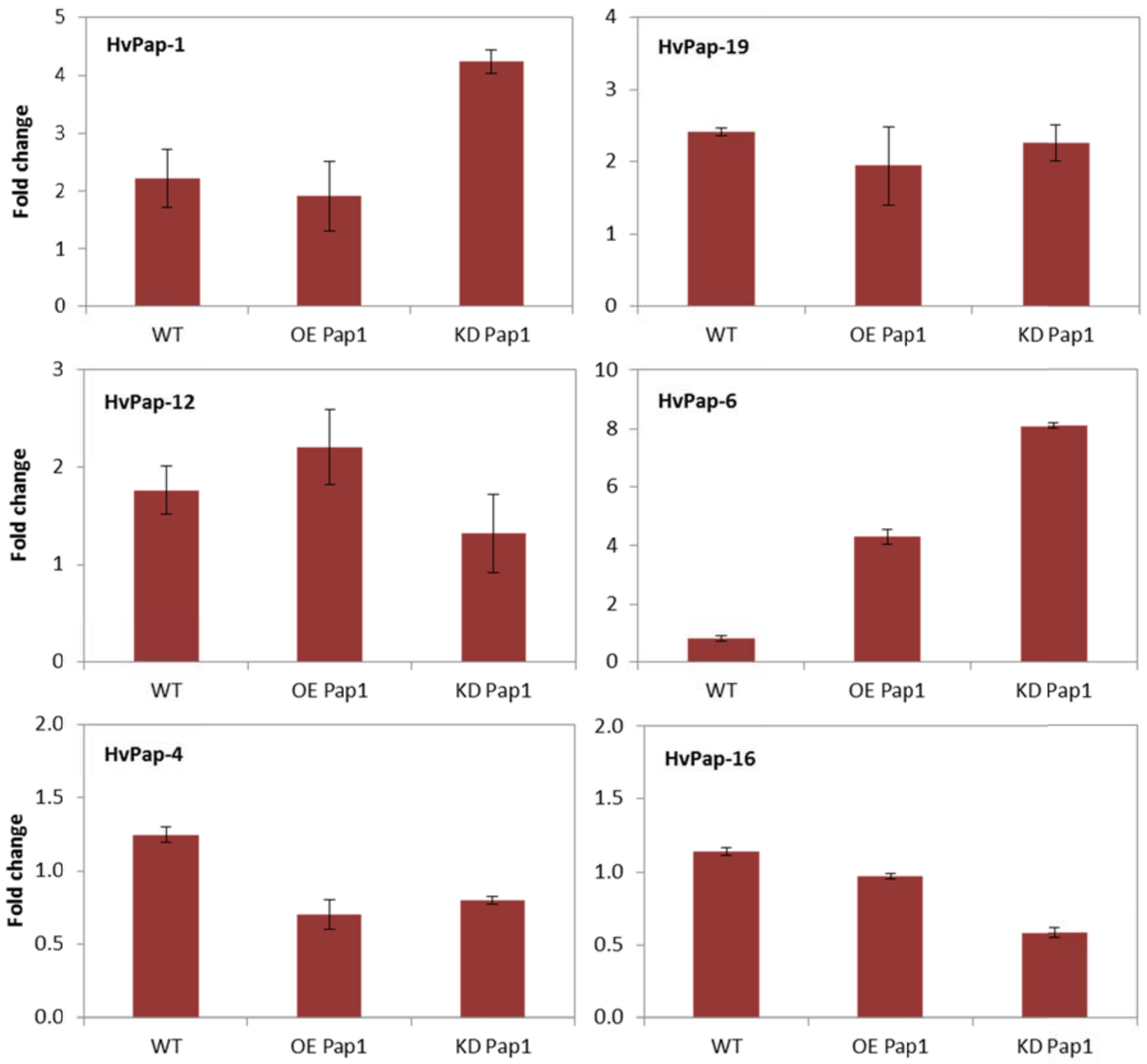

Supplementary Figure 5. Fold change in the expression of C1A CysProt genes (*HvPap-1*, *-19*, *-12*, *-6*, *-4* and *-16*) in transgenic *HvPap-1* overexpressing (OE Pap1, 919) and silencing (KD Pap, 1175) lines, and wild-type (WT) barley plants after 14 d of *T. urticae* infestation, assayed by RT-qPCR. Data were expressed as relative expression to the mRNA levels of C1A CysProt genes in non-treated plants normalized to barley *cyclophilin* mRNA content.

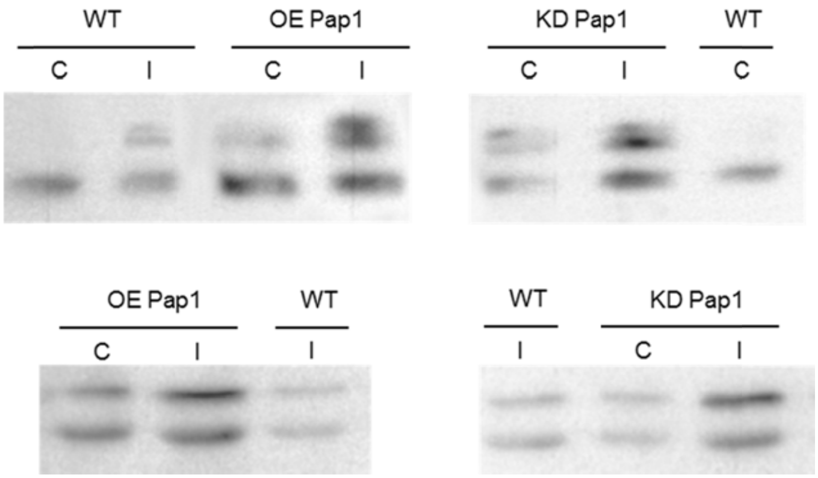

Supplementary Figure 6. Photographs from different gels showing protein accumulation patterns of HvPap-6 in transgenic (OE Pap1 and KD Pap1) and wild-type (WT) barley plants during *T. urticae* infestation assayed by immunoblot. Total protein was extracted from leaves after 14 d of infestation (I) and non-infested leaves (C).

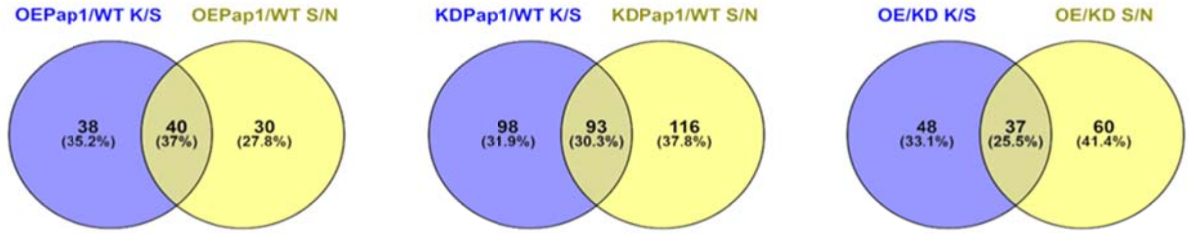

Supplementary Figure 7. Differentially expressed genes (DEGs) in overexpressing (OE Pap1 919) and silencing (KD Pap1 1175) *HvPap-1* transgenic lines. Venn diagrams show shared detected DEGs between SOAP/NOISeq (S/N) and Kallisto/Sleuth (K/S) RNA-seq analyses.

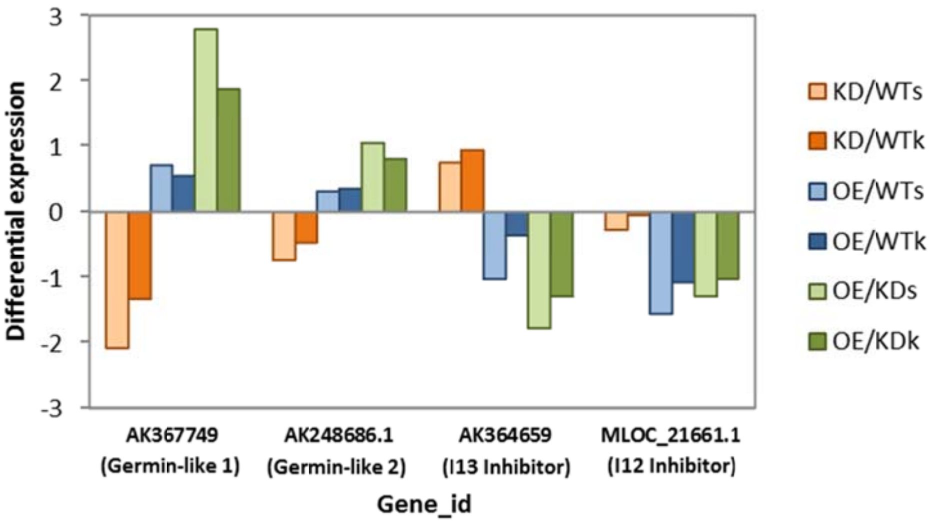

Supplementary Figure 8. Differential RNA-seq expression of selected DEGs between genotypes. Bars represent log2Ratios or b values obtained using the SOAP/NOISeq (s) and Kallisto/Sleuth (k) analyses.

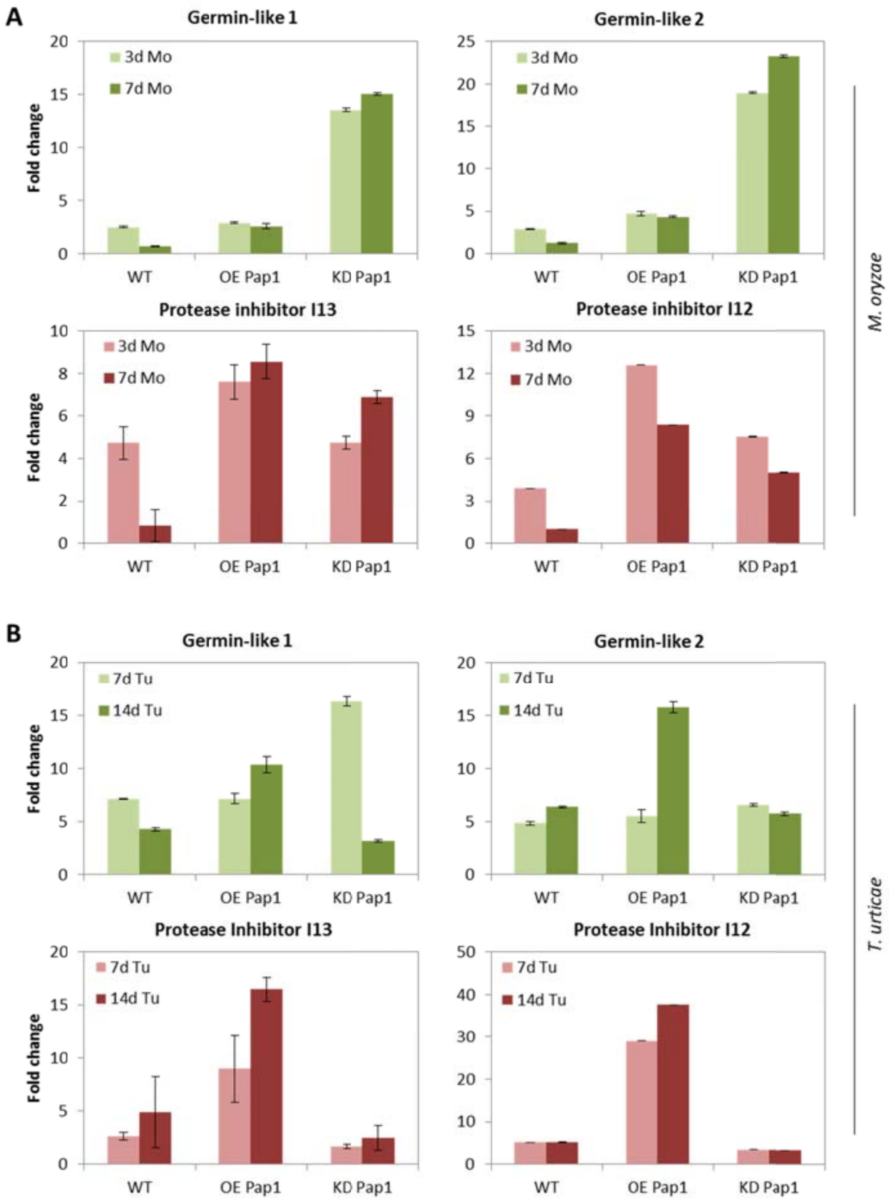

Supplementary Figure 9. Fold change in the expression of germin-like genes and protease inhibitors genes from I12 Bowman-Birk and I13 Potato Inhibitor I families, in transgenic HvPap-1 overexpressing (OE Pap1, 919) and silencing (KD Pap1, 1175) lines, and wild-type (WT) barley plants, assayed by RT-qPCR. Total RNA was extracted form leaves after (A) three (3d) or seven (7d) days of *M. oryzae* infection (light and dark green) or (B) seven (7d) or fourteen (14d) days of *T. urticae* infestation (light and dark red). Data were expressed as relative expression to the mRNA levels of the same genes in non-treated plants normalized to barley *cyclophilin* mRNA content.

1.2 Supplementary Tables

Supplementary Table 1. Primer sequences used for the amplification of barley genes by RT-qPCR assays. *HvPap-4, HvPap-6* and *HvPap-16* genes (cathepsin L-like proteases), *HvPap-1* gene (cathepsin F-like protease), *HvPap-19* gene (cathepsin B-like protease) and *HvCycl* (cyclophilin). *Mo28S-rRNA* gene (*Magnaporthe oryzae* small subunit of ribosomal RNA). *TuRP49* gene (*Tetranychus urticae* Ribosomal Protein 49). *HvI12* (Bowman-Birk) and *HvI13* (Potato Inhibitor I) genes (protease inhibitors). *HvGerm1* and *HvGerm2* genes (germin-like).

**Genes**	**Primers**
HvCycl	forward: 5’-TCCACCGGAGAGGAAGTACAGT-3’<br>reverse: 5’-AATGTGCTCAGAGATGCAAGGA-3’
HvPap-1	forward: 5´-TCCTGGAGTCGATCTTTGGTTTC-3’<br>reverse: 5´-CAAGCATACTGTTGCGGCTTC-3’
HvPap-4	forward: 5´-CCTTGAGAGTCCTTGTTCCCGA-3´<br>reverse: 5´-CCATGTTCGTCGTTTTAACCGA-3´
HvPap-6	forward: 5´-TGCAATTGACGGCAAGAAGA-3´<br>reverse: 5´-TGGATCACCAGGTGATCATTTG-3´
HvPap-12	forward: 5´-ATGTGCGCTATTGCTACCTGC-3´<br>reverse: 5´-CACCTTATTCATGTCTGGCGAA-3´
HvPap-16	forward: 5´-CTGGATCGGTAAGAACTCGTGG-3´<br>reverse: 5´-TGATGGAGGTGCCATCATATGA-3´
HvPap-19	forward: 5´-CACCTTATTCATGTCTGGCGAA-3´<br>reverse: 5´-TGCCCGCTTAATTTGACAGG-3´
Mo28S-rRNA	forward: 5´-TACGAGAGGAACCGCTCATTCAGATAATT-3´<br>reverse: 5´-TCAGCAGATCGTAACGATAAAGCTACTC-3´
TuRP49	forward: 5´-CTTCAAGCGGCATCAGAGC-3´<br>reverse: 5´-CGCATCTGACCCTTGAACTTC-3´
HvI12	forward: 5´- AGGACAAGGCGAGAGGTTTG-3´<br>reverse: 5´- GAAGAACCTGCAGTTGTCGC-3´
HvI13	forward: 5´- CTCACCGTGAATCCTGTCCC-3´<br>reverse: 5´- TTAGCCAATGCGGGGAACTT-3´
HvGerm1	forward: 5´- CGCATCGACTATGCTCCCTT-3´<br>reverse: 5´- TGAAGGTGAATGAGCCCCAC-3´

HvGerm2	forward: 5´- ATGGCATCCTCCCCTACCTT-3´<br>reverse: 5´- CGGCAACACAAAAGTCCTGG-3´

Supplementary Table 2. CysProt amino acid sequences used for specific antibody production.

**Barley proteases**	**Peptides**
HvPap-1	SGFAPSRFKEKPYWIIKN
HvPap-6	IDSEEDYPYKERDNRC
HvPap-16	RLRSYKKVTPPGNEAGLKE
HvPap-19	CQEKKHFSIDAYQVNSDPHD

Supplementary Table 3. List of the top ten significant results obtained from the enrichment gene ontology (GO) analysis of the Biological Process assigned to the DEGs.

Kallisto/Sleuth						
	**GO.ID**	**Term**	**Annotated**	**Significant**	**Expected**	**classicFisher**
**1**	GO:0009052	pentose-phosphate shunt, non-oxidative b...	3	1	0,01	0,01
**2**	GO:0009245	lipid A biosynthetic process	4	1	0,01	0,01
**3**	GO:0042822	pyridoxal phosphate metabolic process	4	1	0,01	0,01
**4**	GO:0042823	pyridoxal phosphate biosynthetic process	4	1	0,01	0,01
**5**	GO:0046493	lipid A metabolic process	4	1	0,01	0,01
**6**	GO:1901269	lipooligosaccharide metabolic process	4	1	0,01	0,01
**7**	GO:1901271	lipooligosaccharide biosynthetic process	4	1	0,01	0,01
**8**	GO:0019566	arabinose metabolic process	5	1	0,02	0,02
**9**	GO:0046373	L-arabinose metabolic process	5	1	0,02	0,02
**10**	GO:0019321	pentose metabolic process	10	1	0,04	0,04
**SOAP/NOISeq**						
	**GO.ID**	**Term**	**Annotated**	**Significant**	**Expected**	**classicFisher**
**1**	GO:0051186	cofactor metabolic process	139	4	0,54	0,002
**2**	GO:0006732	coenzyme metabolic process	112	3	0,44	0,01
**3**	GO:0006637	acyl-CoA metabolic process	3	1	0,01	0,01
**4**	GO:0009052	pentose-phosphate shunt, non-oxidative b...	3	1	0,01	0,01
**5**	GO:0035383	thioester metabolic process	3	1	0,01	0,01
**6**	GO:0008152	metabolic process	7509	35	29,24	0,01
**7**	GO:0042822	pyridoxal phosphate metabolic process	4	1	0,02	0,02
**8**	GO:0042823	pyridoxal phosphate biosynthetic process	4	1	0,02	0,02
**9**	GO:1901564	organonitrogen compound metabolic proces...	875	8	3,41	0,02
**10**	GO:0019566	arabinose metabolic process	5	1	0,02	0,02

Supplementary Table 4. Differentially expressed genes between overexpressing OEPap-1 and silencing KDPap-1 plants obtained by Kallisto/Sleuth method (qval<0.01; b>±1).

target_id	pval	qval	b	BARLEX
**AK360179**	8,13E-23	5,65E-20	4,43	Nucleic acid-binding-like protein
**AK371503**	0,00039246	0,00967949	4,35	DCN1-like protein 4
**MLOC_43997.1**	5,48E-16	1,90E-13	4,02	F-box-like protein
**AK371117**	2,86E-07	2,31E-05	3,70	ATP-dependent zinc metalloprotease FtsH 2
**MLOC_81902.1**	1,39E-07	1,24E-05	3,39	Unknown protein
**MLOC_47391.1**	1,02E-07	9,51E-06	3,38	ATP synthase subunit-like protein
**MLOC_79498.2**	1,48E-82	1,29E-78	3,03	**Cysteine proteinase cathepsin F**
**MLOC_72300.1**	4,06E-05	0,00153465	3,00	GrpE protein homolog
**MLOC_56246.1**	4,29E-06	0,00023329	2,91	Retrotransposon protein, putative, unclassified
**MLOC_80436.1**	0,00020482	0,00573598	2,45	Unknown protein
**MLOC_497.1**	8,32E-07	5,64E-05	2,33	Transposon protein, putative, CACTA, En/Spm sub-class
**AK370563**	2,48E-42	6,15E-39	2,30	Alpha-N-arabinofuranosidase A
**AK251542.1**	1,15E-17	5,10E-15	2,28	Retrotransposon protein, putative, Ty3-gypsy subclass
**MLOC_13731.1**	0,00011963	0,00374678	2,28	BLE2 protein
**AK363961**	1,41E-07	1,25E-05	2,13	Ubiquitin carboxyl-terminal hydrolase
**MLOC_26926.1**	4,85E-08	4,99E-06	2,11	Unknown protein
**AK367749**	8,64E-33	1,36E-29	1,88	Germin-like protein 4d
**AK250005.1**	1,88E-17	7,94E-15	1,71	Ribonuclease 3
**AK374357**	1,02E-05	0,00048168	1,69	Zinc finger protein-like protein
**MLOC_55511.1**	3,43E-06	0,00019318	1,65	Unknown protein
**MLOC_37652.5**	0,0001061	0,00340368	1,58	Esterase/lipase/thioesterase-like protein
**MLOC_33258.3**	5,24E-12	1,17E-09	1,56	FAR1-related sequence 5
**MLOC_57450.1**	0,00022915	0,00625586	1,42	Subtilase
**MLOC_69589.1**	3,69E-08	3,86E-06	1,39	PBSP domain-containing protein
**AK368795**	5,97E-05	0,00212782	1,38	Cytochrome P450 family protein
**MLOC_18260.2**	3,82E-05	0,00147274	1,30	Receptor-like kinase
**MLOC_74854.1**	1,62E-05	0,0007092	1,29	Flowering locus T3
**MLOC_64435.2**	4,16E-06	0,00022808	1,29	Resistance protein, putative
**AK356201**	3,35E-05	0,00131577	1,29	Proline-rich protein
**MLOC_78131.2**	6,25E-07	4,43E-05	1,26	Transposon protein, putative, Mutator sub-class
**AK375581**	3,16E-07	2,51E-05	1,25	Major facilitator superfamily protein
**AK251585.1**	4,30E-07	3,23E-05	1,22	B3 domain-containing protein
**MLOC_13987.1**	1,99E-10	3,41E-08	1,17	ATP-dependent DNA helicase mph1
**MLOC_14895.1**	5,75E-05	0,00206897	1,17	Cytochrome P450 family protein
**MLOC_17984.1**	0,00021738	0,00600052	1,13	Cytochrome P450 family protein
**AK363296**	4,88E-09	6,24E-07	1,12	Chaperone protein dnaJ
**AK372606**	0,00013141	0,00403131	1,11	FAD-binding Berberine family protein
**AK376278**	0,00011506	0,00362563	1,11	Terpenoid cyclases/Protein prenyltransferases superfamily
**AK369808**	5,60E-06	0,00029487	1,10	-
**MLOC_36824.4**	0,00018679	0,00536078	1,07	Serine carboxypeptidase family protein

**MLOC_14999.1**	0,00015418	0,00457623	1,05	Leucine-rich receptor-like protein kinase family protein
**AK252681.1**	6,30E-23	4,75E-20	1,05	Ribonuclease 3
**MLOC_53792.1**	0,00038359	0,0095147	1,04	Lysine decarboxylase-like protein
**AK365122**	6,80E-50	2,36E-46	-1,00	-
**MLOC_44822.1**	1,10E-09	1,59E-07	-1,00	Beta-lactamase-like protein
**MLOC_21661.1**	0,00032614	0,00843921	-1,03	Wound induced protein homolog BBI
**AK363223**	2,03E-05	0,00086853	-1,05	G-type lectin S-receptor-like serine/threonine-protein kinase
**MLOC_66756.1**	3,37E-15	1,08E-12	-1,05	Pyridoxal biosynthesis lyase pdxS
**MLOC_33244.1**	0,00025964	0,00696787	-1,06	Flowering promoting factor-like 1, putative
**MLOC_58847.1**	5,85E-19	2,99E-16	-1,07	60S ribosomal protein L13a-2, putative
**MLOC_62134.5**	8,83E-05	0,00293763	-1,10	FAR1-related sequence 3 LENGTH=851
**MLOC_5129.1**	1,10E-30	1,59E-27	-1,14	60S ribosomal protein L35
**MLOC_74604.1**	0,0001953	0,00551383	-1,15	F-box domain containing protein
**AK360713**	7,12E-05	0,00247875	-1,18	Early light-induced protein
**MLOC_58359.1**	7,96E-07	5,49E-05	-1,21	CsAtPR5
**MLOC_64967.1**	1,63E-24	1,49E-21	-1,21	Eukaryotic aspartyl protease family protein
**AK374103**	1,44E-07	1,27E-05	-1,25	RNA polymerase-associated protein LEO1, putative
**MLOC_53106.1**	3,22E-10	5,22E-08	-1,25	Gibberellin receptor GID1L2
**MLOC_62844.2**	2,17E-66	9,43E-63	-1,26	basic helix-loop-helix (bHLH) DNA-binding superfamily protein
**AK369503**	5,61E-05	0,00203418	-1,29	Glutamate receptor
**AK364659**	2,16E-16	8,33E-14	-1,29	Chymotrypsin inhibitor-2
**MLOC_55770.2**	0,00014885	0,00443698	-1,33	O-methyltransferase
**MLOC_7958.1**	2,03E-05	0,00086853	-1,41	Tetratricopeptide repeat (TPR)-like superfamily protein
**MLOC_18321.1**	1,62E-06	0,00010294	-1,44	Protein of unknown function, DUF642
**MLOC_13047.1**	2,52E-06	0,00014816	-1,46	Leucine-rich receptor-like protein kinase family protein
**MLOC_56456.1**	2,20E-06	0,00013351	-1,49	exocyst subunit exo70 family protein H6
**MLOC_73695.2**	2,70E-05	0,00109902	-1,59	Protein kinase family protein
**MLOC_80345.1**	0,00024517	0,00662037	-1,62	Transposon protein, putative, unclassified
**AK357833**	9,87E-10	1,46E-07	-1,73	Early light-induced protein
**AK355828**	9,28E-08	8,81E-06	-1,73	O-methyltransferase
**MLOC_72140.1**	4,83E-05	0,00177984	-2,17	Lipoxygenase
**AK357115**	1,02E-06	6,75E-05	-2,18	F-box domain containing protein
**MLOC_56951.2**	4,25E-06	0,00023232	-2,28	FBD-associated F-box protein
**MLOC_26429.1**	8,02E-06	0,00040462	-2,43	Transposon protein, putative, ping/pong/SNOOPY sub-class
**AK248248.1**	8,08E-06	0,00040619	-2,72	Early light-induced protein
**MLOC_52920.4**	9,71E-09	1,16E-06	-2,89	H-ATPase
**MLOC_80344.1**	2,12E-75	1,23E-71	-2,95	GRF zinc finger family protein
**MLOC_61767.1**	8,51E-05	0,00284604	-3,08	FAR1-related sequence 3 LENGTH=851
**MLOC_75728.1**	1,14E-05	0,0005305	-3,11	Unknown protein
**AK371482**	1,15E-165	2,01E-161	-3,19	Translation initiation factor eIF-2B gamma subunit
**MLOC_77197.1**	1,95E-10	3,38E-08	-3,21	Transposon protein, putative, Mutator sub-class
**MLOC_7444.1**	6,04E-07	4,31E-05	-3,30	HAT family dimerisation domain containing protein
**MLOC_49726.1**	9,98E-15	3,04E-12	-3,73	Retrotransposon protein, putative, unclassified
**MLOC_3164.1**	3,33E-13	8,14E-11	-3,87	Replication protein A 32 kDa subunit

**MLOC_28203.1**	6,14E-41	1,33E-37	-4,87	Enoyl-CoA hydratase/isomerase family protein, expressed

Supplementary Table 5. Differentially expressed genes between overexpressing OEPap-1 and silencing KDPap-1 plants obtained by SOAP/NOISeq method (log2Ratio>±1; Probability>0.80).

Gene_id	log2Ratio	Probability	BARLEX annotation
**MLOC_56568.1**	10.15	0.94	OJ1005_B10.23 protein
**MLOC_81902.1**	9.91	0.93	Unknown protein
**MLOC_47391.1**	8.86	0.85	ATP synthase subunit-like protein
**MLOC_43997.1**	8.70	0.84	F-box-like protein
**MLOC_26534.1**	8.39	0.80	Wound induced protein
**AK360179**	6.61	0.97	Nucleic acid-binding-like protein
**MLOC_79498.2**	4.49	0.95	**Cysteine proteinase cathepsin F**
**AK251542.1**	3.41	0.90	Retrotransposon protein, putative, Ty3-gypsy subclass
**AK370563**	3.34	0.91	Alpha-N-arabinofuranosidase A
**MLOC_26926.1**	2.98	0.87	Unknown protein
**AK367749**	2.79	0.91	Germin-like protein 4d
**AK250005.1**	2.56	0.91	Ribonuclease 3
**MLOC_55511.1**	2.42	0.87	Unknown protein
**MLOC_23736.1**	2.29	0.82	Unknown protein
**MLOC_33258.3**	2.14	0.87	FAR1-related sequence 5
**MLOC_69589.1**	2.08	0.84	PBSP domain-containing protein
**MLOC_57450.1**	2.02	0.81	Subtilase
**MLOC_74854.1**	1.89	0.87	Flowering locus T3
**AK369808**	1.67	0.86	-
**AK252681.1**	1.59	0.88	Ribonuclease 3
**AK370706**	1.47	0.85	ATP-dependent zinc metalloprotease FtsH 2
**AK248581.1**	1.46	0.80	Beta-glucosidase, putative
**AK251990.1**	1.41	0.85	Cysteine-rich secretory protein
**AK248844.1**	1.41	0.85	Acyl-CoA thioesterase family protein
**MLOC_11817.1**	1.37	0.85	Receptor kinase 1
**MLOC_39318.1**	1.35	0.80	Pathogenesis-related thaumatin-like protein
**MLOC_61601.1**	1.31	0.84	Pyrimidine 5'-nucleotidase
**MLOC_65311.2**	1.27	0.84	Chitinase
**AK250430.1**	1.24	0.86	GDSL esterase/lipase
**MLOC_72157.3**	1.24	0.84	Leucine-rich repeat receptor-like protein kinase family protein
**MLOC_70817.1**	1.20	0.85	Ribulose bisphosphate carboxylase/oxygenase activase
**MLOC_63556.4**	1.15	0.84	CBL-interacting protein kinase 3
**AK360763**	1.13	0.83	-
**MLOC_81131.1**	1.07	0.80	WRKY transcription factor 20
**MLOC_71275.2**	1.06	0.84	Lipoxygenase
**AK248686.1**	1.05	0.84	Germin-like protein 4
**AK250046.1**	-1.00	0.83	Alanine aminotransferase
**AK360133**	-1.00	0.81	cell wall protein precursor, putative

**MLOC_57069.4**	-1.01	0.82	tRNA-dihydrouridine synthase
**AK253095.1**	-1.02	0.83	Proline-rich protein
**MLOC_63089.10**	-1.02	0.80	Asparagine synthetase
**AK250831.1**	-1.02	0.82	Oxidoreductase, zinc-binding dehydrogenase family protein
**MLOC_21619.1**	-1.03	0.83	CASP-like protein
**MLOC_3702.1**	-1.04	0.84	Preprotein translocase subunit SECE1
**MLOC_63184.1**	-1.04	0.81	WRKY transcription factor 10
**AK252852.1**	-1.05	0.84	Proline-rich protein
**AK373369**	-1.05	0.82	RNA polymerase-associated protein LEO1, putative
**AK371226**	-1.05	0.80	Type 1 phosphatases regulator YPI1
**AK365909**	-1.06	0.82	Bifunctional inhibitor/lipid-transfer protein/seed storage 2S albumin superfamily protein
**MLOC_54272.1**	-1.07	0.82	1-aminocyclopropane-1-carboxylate oxidase
**MLOC_56451.1**	-1.07	0.81	Siroheme synthase
**MLOC_64063.2**	-1.08	0.83	Nuclear transport factor 2 (NTF2) family protein with RNA binding (RRM-RBD-RNP motifs) domain LENGTH=460
**MLOC_59790.1**	-1.10	0.83	Eukaryotic aspartyl protease family protein, expressed
**MLOC_12849.1**	-1.11	0.84	Tobamovirus multiplication-like protein
**MLOC_64953.2**	-1.11	0.82	Thioredoxin-like protein
**MLOC_218.1**	-1.14	0.84	cell wall protein precursor, putative
**AK358692**	-1.14	0.80	40S ribosomal protein S8
**MLOC_3743.1**	-1.14	0.81	Peroxidase 1
**MLOC_38648.2**	-1.17	0.83	Concanavalin A-like lectin
**AK369436**	-1.19	0.85	Protein phosphatase 2C containing protein
**AK372602**	-1.21	0.83	Protein of unknown function, DUF538
**MLOC_79958.1**	-1.24	0.86	NADH dehydrogenase [ubiquinone] 1 alpha subcomplex subunit 2
**MLOC_63045.1**	-1.27	0.86	Glycosyl transferase, group 1 family protein
**AK364337**	-1.28	0.82	Cation diffusion facilitator family transporter
**MLOC_57751.1**	-1.28	0.83	cell wall protein precursor, putative
**AK362753**	-1.28	0.81	HXXXD-type acyl-transferase family protein
**MLOC_33244.1**	-1.32	0.80	Flowering promoting factor-like 1, putative, expressed
**AK376140**	-1.32	0.82	Mediator of DNA damage checkpoint protein
**AK252382.1**	-1.35	0.86	Actin associated protein
**AK363562**	-1.35	0.86	UDP-glycosyltransferase
**AK370024**	-1.39	0.83	60S ribosomal protein l35a
**AK365122**	-1.40	0.87	-
**MLOC_66756.1**	-1.44	0.87	Pyridoxal biosynthesis lyase pdxS
**MLOC_78302.1**	-1.51	0.80	Unknown protein
**MLOC_58847.1**	-1.56	0.88	60S ribosomal protein L13a-2, putative, expressed
**MLOC_5129.1**	-1.58	0.88	60S ribosomal protein L35
**AK360713**	-1.61	0.85	Early light-induced protein
**MLOC_64967.1**	-1.68	0.87	Eukaryotic aspartyl protease family protein
**AK251817.1**	-1.69	0.81	Histone H2B
**MLOC_53106.1**	-1.72	0.87	Gibberellin receptor GID1L2
**MLOC_62844.2**	-1.75	0.89	basic helix-loop-helix (bHLH) DNA-binding superfamily protein

**MLOC_7958.1**	-1.83	0.85	Tetratricopeptide repeat (TPR)-like superfamily protein
**MLOC_4531.2**	-1.89	0.84	RNA binding protein-like
**MLOC_4180.1**	-2.04	0.82	Acyl transferase
**MLOC_15383.1**	-2.32	0.86	Eukaryotic aspartyl protease family protein
**AK357833**	-2.45	0.90	Early light-induced protein
**MLOC_6294.1**	-2.64	0.90	GDSL esterase/lipase
**MLOC_26429.1**	-3.29	0.85	Transposon protein, putative, ping/pong/SNOOPY sub-class
**MLOC_61767.1**	-3.34	0.85	FAR1-related sequence 3
**MLOC_80344.1**	-4.22	0.94	GRF zinc finger family protein
**AK371482**	-4.59	0.95	Translation initiation factor eIF-2B gamma subunit
**MLOC_77197.1**	-5.04	0.87	Transposon protein, putative, Mutator sub-class
**MLOC_7444.1**	-8.70	0.84	HAT family dimerisation domain containing protein, expressed
**MLOC_23209.1**	-8.87	0.85	Unknown protein
**MLOC_3164.1**	-9.23	0.89	Replication protein A 32 kDa subunit
**MLOC_75728.1**	-9.41	0.90	Unknown protein
**MLOC_28203.1**	-11.13	0.97	Enoyl-CoA hydratase/isomerase family protein, expressed
